# Targeted anti-tumor synergistic effects of Myc decoy oligodeoxynucleotides-loaded selenium nanostructure combined with chemoradiotherapy on LNCaP prostate cancer cells

**DOI:** 10.32604/or.2023.044741

**Published:** 2023-11-15

**Authors:** ROGHAYEH GHORBANI, MAHMOUD GHARBAVI, ALI SHARAFI, ELHAM RISMANI, HAMED REZAEEJAM, YOUSEF MORTAZAVI, BEHROOZ JOHARI

**Affiliations:** 1 Department of Medical Biotechnology, School of Medicine, Zanjan University of Medical Sciences, Zanjan, Iran; 2 Nanotechnology Research Center, Ahvaz Jundishapur University of Medical Sciences, Ahvaz, Iran; 3 Zanjan Pharmaceutical Biotechnology Research Center, Zanjan University of Medical Sciences, Zanjan, Iran; 4 Department of Pharmaceutical Biotechnology, School of Pharmacy, Zanjan University of Medical Sciences, Zanjan, Iran; 5 Molecular Medicine Department, Biotechnology Research Center, Pasteur Institute of Iran, Pasteur Avenue, Tehran, Iran; 6 Department of Radiology Technology, School of Allied Medical Sciences, Zanjan University of Medical Sciences, Zanjan, Iran

**Keywords:** Chemoradiotherapy, Combination therapy, Decoy oligodeoxynucleotides, Myc transcription factor, Selenium nanoparticle, Prostate cancer

## Abstract

In the present study, we investigated the synergistic effects of targeted methotrexate-selenium nanostructure containing Myc decoy oligodeoxynucleotides along with X-irradiation exposure as a combination therapy on LNCaP prostate cancer cells. Myc decoy ODNs were designed based on the promoter of *Bcl-2* gene and analyzed by molecular docking and molecular dynamics assays. ODNs were loaded on the synthesized Se@BSA@Chi-MTX nanostructure. The physicochemical characteristics of nanostructures were determined by FTIR, DLS, UV-vis, TEM, EDX, *in vitro* release, and hemolysis tests. Subsequently, the cytotoxicity properties of them with and without X-irradiation were investigated by uptake, MTT, cell cycle, apoptosis, and scratch assays on the LNCaP cell line. The results of DLS and TEM showed negative charge (−9 mV) and nanometer size (40 nm) for Se@BSA@Chi-DEC-MTX NPs, respectively. The results of FTIR, UV-vis, and EDX showed the proper interaction of different parts and the correct synthesis of nanoparticles. The results of hemolysis showed the hemocompatibility of this nanoparticle in concentrations less than 6 mg/mL. The ODNs release from the nanostructures showed a pH-dependent manner, and the release rate was 15% higher in acidic pH. The targeted Se@BSA@Chi-labeled ODN-MTX NPs were efficiently taken up by LNCaP cells by targeting the prostate-specific membrane antigen (PSMA). The significant synergistic effects of nanostructure (containing MTX drug) treatment along with X-irradiation showed cell growth inhibition, apoptosis induction (~57%), cell cycle arrest (G2/M phase), and migration inhibition (up to 90%) compared to the control. The results suggested that the Se@BSA@Chi-DEC-MTX NPs can potentially suppress the cell growth of LNCaP cells. This nanostructure system can be a promising approach for targeted drug delivery and chemoradiotherapy in prostate cancer treatment.

## Introduction

In 2020, prostate cancer (PC) was the second most common cancer and the fifth leading cause of cancer death in men [1]. Prostate-specific antigen (PSA) can be effectively used in early-stage the diagnosis of prostate cancer. PC can be treated with surgery and X-ray irradiation therapy [2]. But, at the late stages, it can metastasize to other tissues and cause a major problem for treatment [3]. Death from prostate cancer most often happens when cancer has spread (metastasized) to other organs in the body. The current treatments for metastatic PC are hormone therapy, chemotherapy, immunotherapy, and Radium-223 [4]. However, over time, cancer cells become resistant to these treatments and turn into hormone-resistant prostate cancer [5]. There is no effective treatment for this type of PC that is responsible for most patients’ deaths. Therefore, the development of new treatments for metastatic prostate cancer using targeting strategies is required.

Mutations in genes involved in the cell signaling pathways can affect cell death or survival *via* molecular regulation of cell cycle and apoptosis [6]. The main molecular signaling pathways of prostate cancer are the androgen receptor (AR) mediated signaling pathway, NF-κB, PI3K/AKT, MAPK, TGF-β/SMAD, JAK/STAT, and Wnt signaling pathway [7]. It is noteworthy that Myc transcription factor mediates tumorigenic functions of several oncogenic signaling pathways such as Wnt, Ras, and phosphatidylinositol 3-kinase/Akt [8]. Myc transcription factor has an important role in regulating cell metabolism, protein production in cells, cell-cycle progression, mitochondrial function, and stem cell self-renewal [9]. The Myc oncogene, a downstream target of PI3K/AKT pathway, is commonly upregulated in many types of cancers [10]. Activation of Myc proto-oncogene is one of the first molecular changes in prostate cancer, so that, it may be a key molecular marker in the early diagnosis of this disease. c-Myc mRNA is raised in most prostate cancers even in early stages and grades (e.g., Gleason score 6); so, it plays an important role in the initiation of PC [11]. In benign prostatic epithelial cells, TGF-β inhibits proliferation and induces apoptosis through SMAD-mediated changes in c-Myc (down-regulation) and cyclin-dependent kinase (G1 arrest) [12]. It revealed that c-Myc transcription factors have a key role in PC [13]. So, transcription factors (TFs) can be important and putative targets for cancer treatment due to their functions in many oncogenic signaling pathways [14].

Several strategies, such as antisense oligonucleotides [15], siRNAs and microRNAs [16], small molecule protein/protein interaction inhibitors [17], and the compounds that specifically inhibit Myc binding have been used to block the function of Myc transcription factor. One type of oligonucleotide-based drugs is short double-stranded DNA molecules called transcription factor ODNs decoys (TFDs). These synthetic decoys bind to the specific genome binding site of TFs and prevent their interaction with their promoter region, so, competitively inhibit their activity and suppress the induction of gene transcription [18,19]. Compared to other DNA or RNA-based gene silencing technologies, such as RNA interference (RNAi) technology (siRNAs) which contain specific sequences that are only complement to a single target mRNA, TFDs are designed to inhibit several genes involved in specific pathways at the pre-transcriptional level [14].

One of the limitations to using decoy oligodeoxynucleotides as a drug is the low ability to cross cell membranes. To overcome this problem in order to efficiently transfer TFDs into the cell, nanotechnology has been suggested. Thus, the design of novel non-viral gene delivery carriers has been considered in cancer gene therapy strategies [20]. There are several nanoscale drug carriers such as metal nanoparticles (NPs), liposomes, ceramic materials, and polymeric micelles [21].

Chemotherapy along with surgery and radiotherapy usually used in the advanced stages of cancer [22]. Methotrexate (MTX) (analog of folate) as a chemotherapeutic drug and as a ligand-target is effectively administrated to treat cancer [23,24]. However, the use of MTX has limitations such as poor solubility, toxic side effects, and nonspecific drug delivery to non-target tissues [25]. Some studies have shown that LNCaP and PC3 cancer cells do not express the folate receptor on their membrane, but prostate-specific membrane antigen (PSMA) is highly expressed in these cancer cells and malignant prostate tissue [26,27]. It is suggested that the uptake of folate-linked NPs in LNCaP cells may be mediated by PSMA [28]. So, methotrexate-targeted NPs can enter into LNCaP cells through PSMA receptors. Various nanoscale drug delivery systems such as selenium nanoparticles (SeNPs) have been developed to reduce the toxic side effects and improve the effectiveness of these drugs [29]. SeNPs have been used as anticancer drug/gene vehicles [30] due to well biocompatibility and anti-cancer activity [31]. Previous studies are being conducted to investigate the anti-cancer effects of selenium nanoparticles against various types of cancers [32,33]. These nanoparticles also play an important role in combating diseases by reducing drug side effects, regulating thyroid gland function, and ensuring the proper functioning of the immune system [34]. On the other hand, bovine serum albumin (BSA) is an excellent biomolecule for nanoparticle surface modification due to its low cost, biocompatibility, stability, and non-interference in biological reactions [35]. Chitosan (CS) as a renewable, sustainable, and cost-effective natural polymer, is used for surface modification of nanoparticles to increase the biocompatibility and drug delivery capabilities of metal nanoparticles [36].

X-ray irradiation exposure is one of the most effective and important methods of tumor treatment to prevent tumor recurrence and prolong the survival of patients. Some studies showed that metal-based nanoparticles usually exhibit chemical inertness in cellular and subcellular systems and may play a role in radio sensitization and synergistic cell-killing effects for radiation therapy [37,38]. In recent years, combination therapies have become more prevalent by reducing drug toxicity, suppressing multidrug resistance, and sensitizing cancer cells to radiotherapy and chemotherapy through different mechanisms such as using specific antisense oligonucleotides of different factors in cancer cells [39,40].

The present study investigated the synergistic effects of targeted methotrexate-selenium nanostructure containing Myc decoy oligodeoxynucleotides along with X-irradiation exposure and chemotherapy as a combinational therapy on LNCaP prostate cancer cells.

## Materials and Methods

### Reagents and materials

Bovine serum albumin (BSA) (CAS 9048-46-8) and Chitosan (CAS 9012-76-4) were purchased from Sigma-Aldrich (Sigma, USA). Methotrexate sodium was gifted by Zahravi Pharmaceutical Company of Iran (Tabriz, Iran). MTT (57360-69-7), FBS (ES-020-B), Trypsin-EDTA (T3924), Sodium selenite (Na_2_SeO_3_) and Penicillin−streptomycin (P4333) obtained from Sigma Aldrich Co. (St Louis, MO, USA). PBS was prepared in the laboratory. Dimethyl sulfoxide (DMSO) (AMT116743) was obtained from Merck (Merck Co., USA). RPMI-1640 medium (Z11030-500) was provided by Zistpajooh (Zistpajooh Co., Iran). Annexin V-FITC/PI kit (APOAF) (Sigma, USA), cell culture plates (SPL Life Sciences South Korea) were also provided. Decoy and Scramble ODNs were synthesized by Bioneer Inc. (Daejeon, Korea). LNCaP cell line was provided by Pasteur Institute of Iran (Tehran, Iran).

### Cell culture

LNCaP cell line is routinely cultured in RPMI 1640 medium supplemented with 10% FBS, 100 units/mL penicillin, and 100 µg/mL streptomycin at 37°C in a humidified atmosphere with 5% CO_2_ incubator.

### Design and synthesis of Myc decoy and scramble ODNs

21-mer phosphorothioate (PS) modifications sense and antisense oligodeoxynucleotides strands of Myc decoy (DEC) and scramble (SCR) as the mutant negative control were designed based on the binding site of Myc transcription factor in the human *Bcl-2* gene and synthesized. Both strands were dissolved in TE buffer (pH 8.0). The annealing was carried out by heating to 90°C for 10 min and then cooled slowly down at room temperature. All double-stranded ODNs were quantitated by NanoDrop™ spectrophotometry and held at 4°C. In the sequences of ODNs, the core binding site, PS modifications at 3′ and 5′ ends (for enhanced stability), and three mutations (in scramble ODNs sequences) are shown in boldface, stars, and italics/underlined, respectively. ODNs were labeled by Cy3 fluorescent dye (Labeled ODNs) at the 3′ termini for tracking of them inside the cells.

DEC ODN sequences:

F [5′T*TGGCAC**CACGTG**GTGGCGA*G3′]

R [5′A*ACCGTGGTGCACCACCGCT*C3′].

SCR ODN sequences:

R [5′T*TGGCAC*A*A*AT*TGGTGGCGA*G3′]

F [5′A*ACCGTGTTTAACCACCGCT*C3′].

### Protein modeling and molecular docking on designed ODNs

Homology modeling was performed by SWISS-MODEL using the amino acid sequence of human Myc protein (retrieved from Uniprot ID: P01106) [41]. The three-dimensional models of the DNA sequences (decoy and scramble) were generated using the 3DNA-Driven DNA Analysis and Rebuilding Tool (3D-DART) web server [42]. To investigate the Myc-ODNs interactions, Myc was docked into the three dimensional B-form of DEC and SCR using HADDOCK (High Ambiguity Driven protein-protein Docking) web server (version 2.2) [43]. The analysis and visualization of the final docked complexes were processed with Ligplot+ and PyMol, respectively [44,45].

### Molecular dynamics (MD) simulation on designed ODNs

The Myc/DEC and Myc/SCR complexes subjected to MD simulation in order to analyze the structure stabilities. The simulation was performed in Gromacs 2020.3 using AMBERff99SB-ILDN force field [46,47]. The system was defined in a rectangular box of transferable intermolecular potential with 3 points (TIP3P) water molecules that neutralized using Na^+^/Cl^−^ ions. Each system was initially subjected to 5000 steps of steepest-descent of energy minimization, where the maximum force was set to 1000 kJ·mol^−1^·nm^−1^. Then, systems equilibrated into NVT (constant number of particles, volume, and temperature) and NPT (constant number of particles, pressure, and temperature) conditions at temperature (300 K) and pressure (1 bar) using Vrescale and Parrinello-Rahman pressure coupling method, respectively. Finally, molecular simulation performed for 50 ns with a time step of 2 fs. The output trajectories were analyzed in terms of root mean square deviation (RMSD), the radius of gyration (RoG), root mean square fluctuation (RMSF), and hydrogen bond profile using Gromacs inbuilt tools.

### Synthesis of nanostructures

Synthesis of BSA-modified SeNPs: BSA-coated selenium nanoparticles (Se@BSA NPs) were synthesized with sodium selenite (Na_2_SeO_3_) and BSA in a two-step reaction. Specifically, SeNPs were synthesized by dissolving 200 mg of BSA and 5 mg of sodium selenite in 5 mL of diH_2_O (Milli-Q system, EMD Millipore, Billerica, MA) and stirring the resulting solution for 1 h at 120°C [48]. After the synthesis of SeNPs, the color of the solution turned dark orange. Se@BSA NPs were synthesized by adding 500 µL of BSA (20 mg/mL) to SeNPs and stirring for 12 h. The synthesized nanoparticles were collected by centrifugation (11,000 rpm at 15 min) and were washed at least twice with diH_2_O.

Synthesis of Se@BSA@Chi NPs and Se@BSA@Chi-MTX NPs: 300 µL chitosan (Chi) solution (6.7 mg/mL) was used to make Se@BSA@Chi NPs by adding to Se@BSA NPs and stirring. On the other hand, 500 µL MTX (2.5 mg/mL) activated with NHS and EDC added to Se@BSA@Chi NPs solution to make Se@BSA@Chi-MTX NPs.

Synthesis of Se@BSA@Chi-DEC NPs and Se@BSA@Chi-SCR NPs: The Se@BSA NPs were dispersed in DNase-/RNase-free water and stirred with ODNs (DEC OR SCR)/Chi solution for 12 h to form Se@BSA@Chi-ODNs (DEC/SCR) complex.

**Figure S1:**
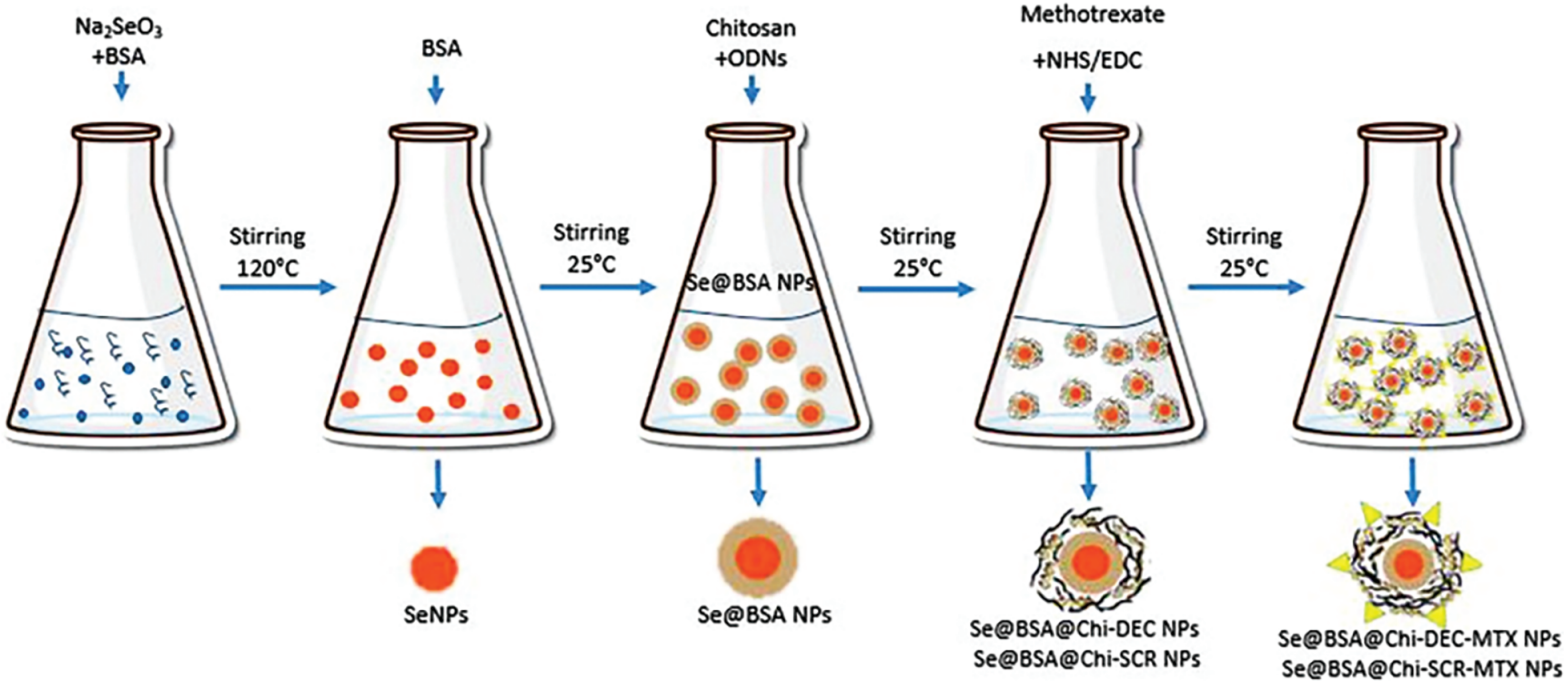
The schematic mechanism for synthesis of all nanostructures used in the current study.

Synthesis of Se@BSA@Chi-DEC-MTX NPs and Se@BSA@Chi-SCR-MTX NPs: The synthesis of Se@BSA@Chi-DEC-MTX NPs and Se@BSA@Chi-SCR-MTX NPs was performed by adding 500 µL MTX (2.5 mg/mL) activated with NHS and EDC to the Se@BSA@Chi-DEC NPs and Se@BSA@Chi-SCR NPs (Suppl. Fig. S1).

### Physicochemical characterization

### FTIR and UV-Vis characterization of NPs

Fourier transform infrared (FTIR) spectroscopy of all synthesized nanoparticles and free DEC ODNs, SCR ODNs, Chi, BSA and MTX were recorded using FTIR spectroscopy over the range of 400–4000 cm^−1^ spectra to distinguish chemical groups. Approximately one drop prepared sample mixed with 100 mg potassium bromide (KBr) under pressure of 10 N. In addition, the absorption spectrum of the nanoparticles and the mentioned samples examined by UV-Vis spectrophotometer in the wavelength range of 200–500 nm to determine the constituents of nanoparticles (ODNs, MTX, BSA, Chi and Se).

### Particle size and z-potential analysis of NPs

Hydrodynamic size (Z-average), size distribution (polydispersity index) and *z*-potential of the NPs were characterized by DLS (Malvern Instruments, Worcestershire, UK, Nano ZS model) at 25°C. All of the NPs were dispersed in ultrapure water in a clean Malvern sample vial to achieve a UV level of 0.07 ± 0.02 units at 633 nm.

### EDX analysis of NPs

For structural or chemical properties analysis of nanoparticle samples, NPs were subjected to elemental composition analysis using an energy dispersive X-ray spectroscopy (EDX) microanalysis system (MIRA3TESCAN-XMU electron microscope).

### TEM analysis of NPs

Transmission electron microscope (TEM) was used for determining of the size and morphology of the synthesized nanoparticles (SeNPs and Se@BSA@Chi-DEC-MTX NPs) using a Philips EM208S 100 KV electron microscope.

### ODNs release experiment from NPs

In this experiment, the sample and separate (SS) method [49] was used to assess the release behavior of decoy and scramble ODNs from the Se@BSA@Chi-SCR, Se@BSA@Chi-SCR-MTX, Se@BSA@Chi-DEC, and Se@BSA@Chi-DEC-MTX NPs. A defined amount of decoy and scramble ODNs-loaded nanoparticle suspended in 1.5 mL of phosphate buffer solution (pH 5.8 and 7.4). Then they were shaken at 120 rpm in an incubator shaker for 72 h. At predefined time intervals, release samples were extracted from the centrifuged mixture. An equal amount of the released media was replaced. The concentrations of released ODNs were determined by spectrophotometry at 260 nm wavelength using the NanoDrop (Wilmington, USA).

### In vitro hemolysis assay

Hemolysis assay was performed using healthy and fresh human blood. The blood was collected in tubes with heparin and the erythrocytes collected by centrifugation at 4000 rpm for 5 min, and then washed three times with phosphate buffered saline at pH 7.4. The purified RBC resuspended in isotonic PBS until it diluted to 10% of its initial concentration. The various nanostructure groups were prepared in PBS buffer with 0.75, 1.5, 3, 6, 12, 24 mg/mL concentrations. The 1% SDS and PBS were used as a positive control (100% hemolysis) and a negative control (0% hemolysis), respectively. 200 µL of RBC were added to 500 µL of the positive control, negative control and nanoparticle dispersions. The tubs were incubated for 4 h at 37°C and after incubation, the percentage of hemolysis was measured by microplate reader analysis of the supernatant at 540 nm absorbance after centrifugation at 4000 rpm for 10 min. This experiment repeated three times, and the percentage of hemolysis calculated as:

$$\text{Hemolysis}\%=\frac{\text{A treated sample}-\text{A negative control}}{\text{ A positive control}-\text{A negative control}}\times 100$$

In this equation, A treated sample, A negative control and A positive control are representative of mean absorbance of the sample, negative control, and positive control, respectively.

### Nanostructure cellular uptake assay

LNCaP cells seeded in 12-well plates at a density of 6 × 10^4^ cells per well and cultured in a 500 μL complete medium. After 24 h incubation at 37°C in 5% CO_2_, the medium was removed and 500 μL of optimum medium containing Se@BSA@Chi-MTX NPs (25 nM) as a negative control group and Se@BSA@Chi-labeled ODNs-MTX NPs formulation with different concentrations (25, 50, 100, 200 nM of labeled ODNs) as positive control were added to each well. After treatment for 24 h, the cells were washed three times with PBS solution and then were harvested by trypsinization and centrifuged at 1000 rpm for 5 min, suspended in 500 μL PBS. They were recorded using flow cytometry (BD Biosciences, San Jose, CA, USA) and analyzed by FlowJo v7 software (Tree Star, Ashland, OR, USA).

### Cell viability assay

LNCaP cells were seeded in 96-well plates at a density of 1.5 × 10^4^ cells/well containing 200 µL of complete medium and were allowed to grow overnight at 37°C in 5% CO_2_. Then, the cells were treated with various nanostructures (SeNPs, Se@BSA NPs, Se@BSA@Chi NPs, Se@BSA@Chi-MTX NPs, Se@BSA@Chi-SCR NPs, Se@BSA@Chi-DEC NPs, Se@BSA@Chi-SCR-MTX NPs, Se@BSA@Chi-DEC-MTX NPs) with different concentrations based on Myc decoy ODNs (25, 50, 100 and 200 nM). 24 h after treatment, 20 μL/well MTT solution (5 mg/mL) was added to each well and the plate was incubated at 37°C for 4 h. After removing the MTT solution, dimethylsulfoxide (DMSO) was added (150 μL/well) to each well. Subsequently, the plate was shaken for 5 min. Finally, the absorbance values were detected by a micro plate reader at a wavelength of 570/630 nm.

### Cell cycle assay

LNCaP cells were seeded in 12-well plates at a density of 6 × 10^4^ cells/well containing 500 µL of complete medium. The plates were incubated overnight at 37°C in 5% CO_2_. Then, the cell treatments were carried out with different types of nanostructures (Se@BSA@Chi NPs, Se@BSA@Chi-MTX NPs, Se@BSA@Chi-SCR NPs, Se@BSA@Chi-DEC NPs, Se@BSA@Chi-SCR-MTX NPs, Se@BSA@Chi-DEC-MTX NPs) based on Myc decoy ODNs concentration (100 nM). The treated cells were detached after 24 h incubation by trypsin (0.05% trypsin/EDTA) and pelleted by centrifuge (1200 rpm for 3 min). After dispersing cell pellets in 50 μL PBS, cell fixation was performed using 70% ethanol. The obtained fixed cells were centrifuged, and alcohol removed. Then, the cells were treated with 1 mL PI (propidium iodide) Master Mix solution (40 μL PI, 10 μL RNase, 950 μL PBS) and incubated at room temperature for 30 min. The data was recorded by flow cytometry FACSCalibur (BD Biosciences, San Jose, CA) and analyzed with FlowJo v.7 software (Tree Star, Ashland, OR). The obtained data was reported as the cell population percentage in each phase of the cell cycle.

### Cell apoptosis assay

LNCaP cells were seeded in 12-well plates at a density of 6 × 10^4^ cells/well containing 500 µL of complete medium. They were allowed to grow at 37°C in 5% CO_2_ overnight. Then, cell treatments were performed with different types of nanostructures (Se@BSA@Chi NPs, Se@BSA@Chi-MTX NPs, Se@BSA@Chi-SCR NPs, Se@BSA@Chi-DEC NPs, Se@BSA@Chi-SCR-MTX NPs, Se@BSA@Chi-DEC-MTX NPs) based on Myc decoy ODNs concentration (100 nM). 24 h after treatment, the cells were harvested and washed with PBS. They were stained with Annexin V-FITC and PI according to the manufacturer’s protocol. Data acquisition and analysis were conducted via flow cytometry FACSCalibur (BD Biosciences, San Jose, CA) and FlowJo software (Tree Star, Ashland, OR).

### Wound healing (scratch) assay

LNCaP cells were seeded at a density of 4 × 10^4^ cells/well in 12-well plates and incubated at 37°C, 5% CO_2_. At approximately 70% confluence, artificial wounds were created by scratching the cell layer with a 200 μL sterile pipette tip. Then, the cells were treated with different types of nanostructures (Se@BSA@Chi-MTX NPs, Se@BSA@Chi-SCR-MTX NPs, Se@BSA@Chi-DEC-MTX NPs) based on Myc decoy ODNs concentration (100 nM). Wound gap closure was photographed at times 0 and 96 h. The migration inhibition percentages were quantified using ImageJ (v1.52; NIH) software. The experiments performed in triplicates.

### X-irradiation treatment

All of the above tests (MTT, cell cycle, apoptosis, and scratch tests) were performed once more for radiotherapy. Briefly, the LNCaP cells were seeded, trypsinized, resuspended, and dispensed in different culture plates depending on the experiment requirement. The cell treatments were carried out with different nanostructures (SeNPs, Se@BSA NPs, Se@BSA@Chi NPs, Se@BSA@Chi-MTX NPs, Se@BSA@Chi-SCR NPs, Se@BSA@Chi-DEC NPs, Se@BSA@Chi-SCR-MTX NPs, Se@BSA@Chi-DEC-MTX NPs) with different concentrations based on Myc decoy ODNs related to each test. 6 h after treatment, subsequent to removing the treatment medium and replacing it with a fresh complete medium, the cells were exposed to a 2 Gy fractionated X-ray irradiation and incubated at 37°C in 95% air/5% CO_2_ until doing the test.

### Statistical analysis

Data analysis performed by using Graphpad Prism 8.0 software and expressed as mean ± standard deviation. Significance was calculated by one-way and two-way analysis of variance for multiple (>2) groups. *p* < 0.05 was considered significant; *p < 0.05; **p < 0.01; ***p < 0.001; and ****p < 0.0001. All experiments carried out in triplicates.

## Results

### Structure of the bHLH domain of Myc protein and molecular docking

Homology modeling of Myc protein by SWISS-MODEL provided a model that belongs to the basic helix–loop–helix zipper (bHLHZip) domain at C-terminal of Myc. Protein conformation of the bHLH domain of Myc (residues 348–439) was based on the coordinates drawn from the Protein Data Bank (PDP ID: 6G6k.A) [50]. The template was the crystal structures of Human MYC: MAX bHLHZip complex at a resolution of 1.35 Å with high sequence identity (100%) and low coverage (0.21).

**Figure 1:**
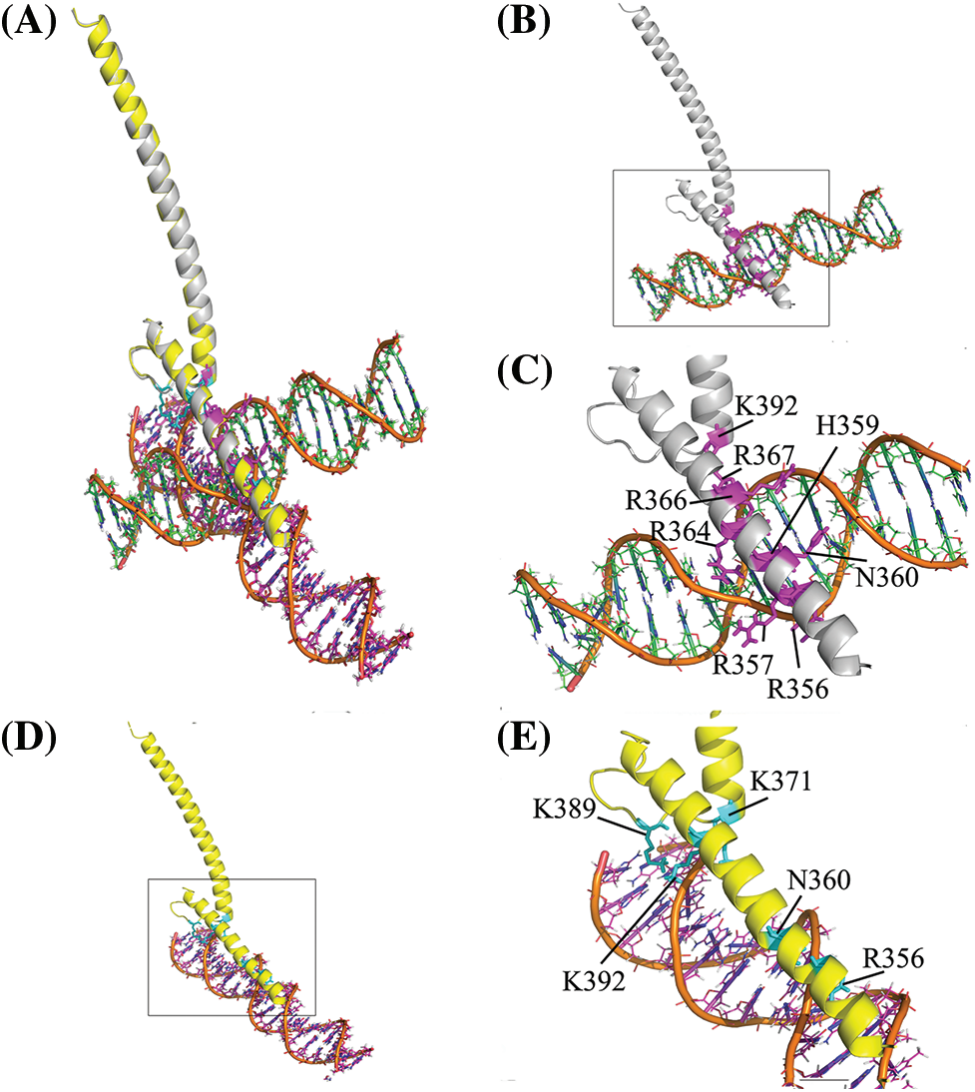
Structural representation of the bHLH domain of Myc-ODNs complexes. (A) The aligned form of complexes to compare the position of each ODN. The DEC and SCR are displayed in orange-green and orange-magenta elements, respectively. (B) Complex of Myc/DEC (Myc in gray color). (C) The zoom view of Myc/DEC binding residues. (D) Complex of Myc/SCR (Myc in yellow). (E) The zoom view of Myc/SCR binding residues. The binding residues depicted in stick format in magenta and cyan in DEC and SCR complexes, respectively.

Molecular docking was performed by designating the Myc basic region with residues K355, R356, H359, E363, and R367 as active residues. But, passive residues were automatically defined around active residues. The complexes of decoy and scramble ODNs with the bHLH domain of Myc were analyzed to find their interaction pattern. The results indicated a distinctively interaction mode of Myc protein with the decoy and scramble ODNs. Likewise, Ligplot+ analysis showed various H-bonds in Myc/DEC and Myc/SCR, 8 and 5 H-bonds, respectively. The aligned complexes of Myc/DEC and Myc/SCR as well as their interactive conformation in detail were depicted in Figs. 1A–1E.

### Molecular dynamics simulations of complexes

**Figure 2:**
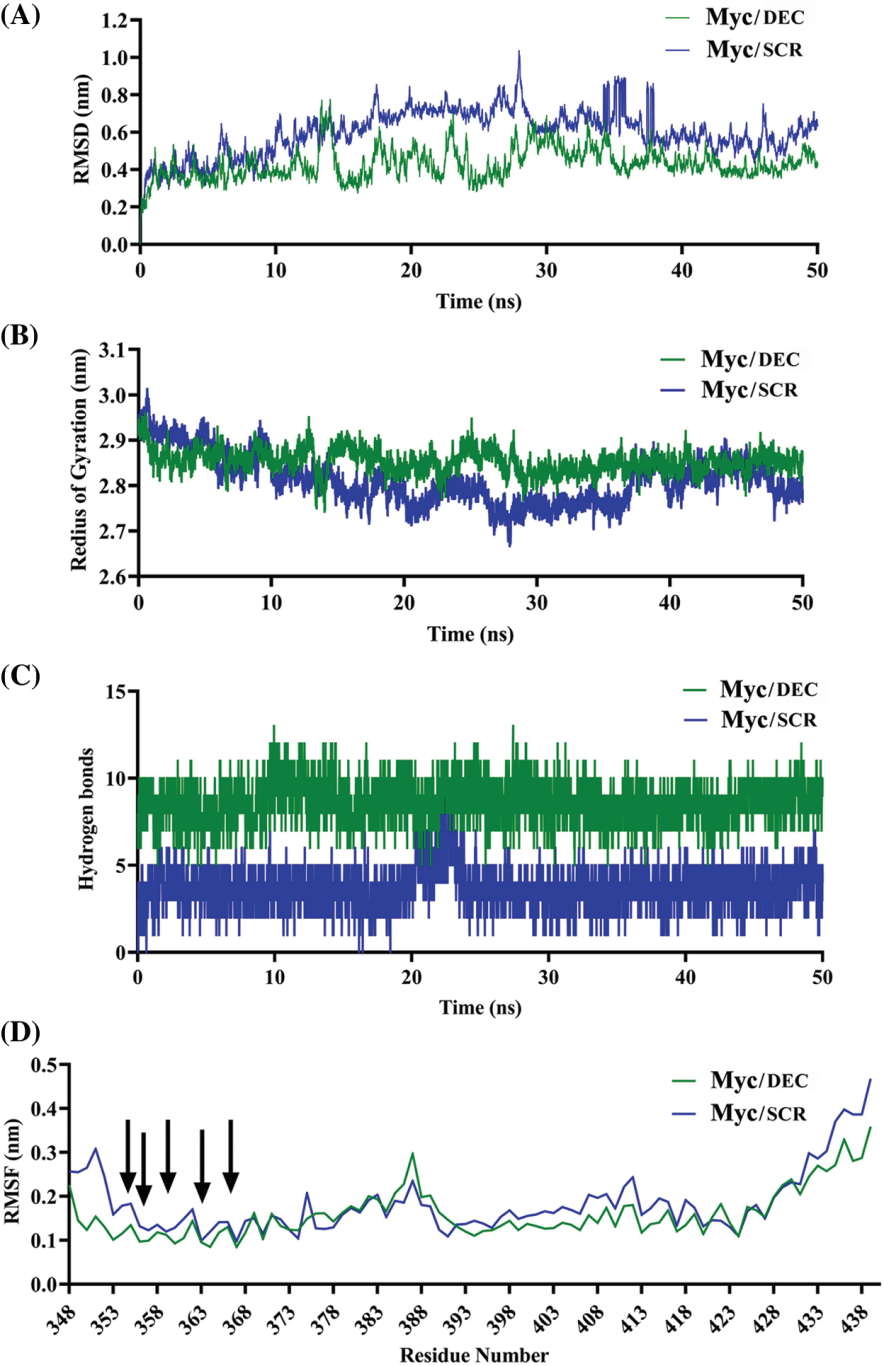
Comparative MD plots of Myc/DEC and Myc/SCR complexes. (A) RMSD was calculated through the least square fitting of backbone atoms, (B) Radius of gyration, (C) Hydrogen bonds between either Myc and DEC or SCR, and (D) RMSF of Myc residues were evaluated during 50 ns MD simulations trajectory to investigate stability and fluctuation of Myc/DEC and Myc/SCR complexes.

Dynamic behavior and stability of Myc protein in complex with either DEC or SCR were evaluated by time-dependent properties during simulations. It observed that Myc bHLH domain remained more stable in complex with DEC than SCR. RMSD value of Myc/DEC showed fewer fluctuations ranging from 0.2–0.4 nm, whereas Myc/SCR was characterized by higher continuous RMSD fluctuations ranging from 0.4–0.6 nm (Fig. 2A).

The radius of gyration, calculated for alpha (α) carbon atoms of protein *vs*. time, displays compactness of protein. The average RoG score for Myc/DEC and Myc/SCR were 2.95 and 2.86 nm, respectively. RoG plot indicated a relatively steady state in globularity for Myc/DEC, though Myc/SCR showed more fluctuations throughout the simulation (Fig. 2B). More variations in RoG of Myc/SCR could indicate that point mutation in the binding site of DNA can cause structural destabilizing effects leading to less binding affinity of SCR to Myc.

To find preserving of the binding affinity and stability of Myc/DEC and Myc/SCR, hydrogen bonds (H-bonds) were evaluated throughout 50ns of simulations. The evaluation results showed that Myc/DEC complex contained relatively constant number of H-bonds (7–10) during the simulation, whereas the number of H-bonds in Myc/SCR was more variable (2–7) (Fig. 2C).

RMSF calculates the average deviation of protein residues over time from the reference position (initial structure). RMSF plot indicated relatively similar residue fluctuation profile for Myc/DEC and Myc/SCR with an average RMSF of 0.18 and 0.25 nm, respectively (Fig. 2D). The maximum fluctuation was seen at 385–413 positions. The residues involved in DNA binding namely, K355, R356, H359, and E363 exhibited lower fluctuations in Myc/DEC complex as compared to Myc/SCR, though R367 showed a similar RMSF value.

### Physicochemical characterization of prepared nanostructures

BSA protein contains 17 disulfide bonds, when exposed to 121°C, the disulfide bonds are broken, the protein is degraded, and more -SH groups are formed. By forming -SH and -OH groups, Se (IV) can be reduced to Se (0), as evidenced by a change in color from clear white to clear dark orange. The color formation in the reaction mixture of selenium nanoparticles is caused by surface plasmon resonance excitations [51].

### FTIR analysis showed interactions between different substances in nanostructures

**Figure 3:**
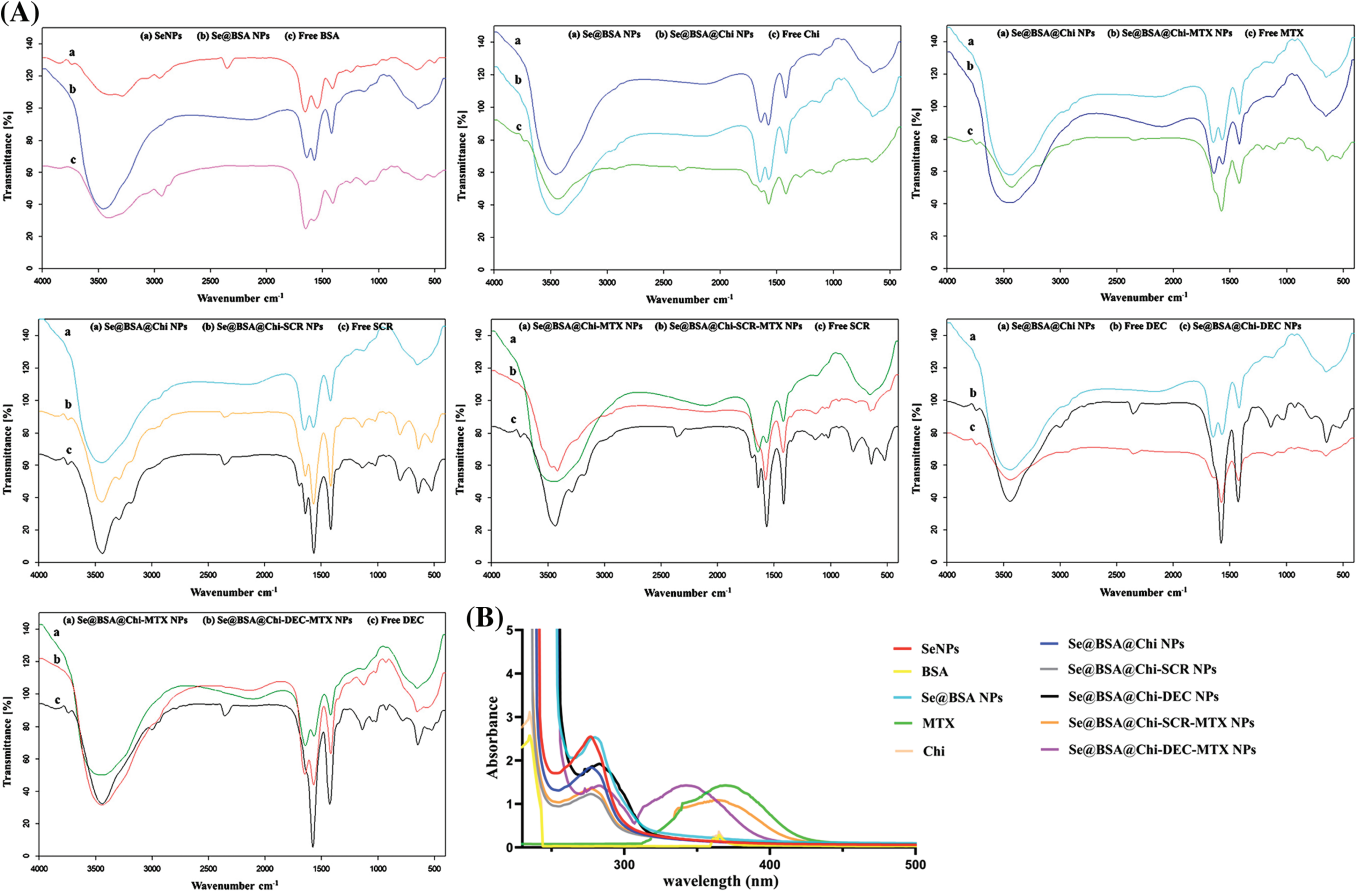
(A) The FTIR spectrum of synthesized different formulations of nanostructures showed the successful synthesis of various formulations. (B) UV-Vis spectra of different formulations of prepared nanostructures and materials participating in their production.

In order to confirm that the different formulations of nanoparticles were synthesized properly, FTIR spectroscopic analysis was performed (Fig. 3A). The FTIR spectrum of BSA was characterized by the amide-I and amide-II bands occurring at 1600–1700 cm^–1^ (mainly C=O stretch) and near 1500 cm^–1^ (C–N stretch coupled with N–H bending mode), respectively [52]. Also, α-helix, β-sheet, and random coil structures in BSA structure correspond with 1650–1658, 1610–1640, and 1640–1658 cm^–1^ peaks, respectively [53]. The FTIR spectrum of BSA recorded in the presence of SeNPs showed that the amide-I and amide-II peaks of BSA shifted from 1650 to 1635 cm^–1^ and from 1550 to 1530 cm^–1^, respectively. The changes in the peak positions can indicate the secondary structure modification of BSA by SeNPs. Moreover, the amide-I peak has shifted from 1650 to 1635 cm^−1^, which provides information regarding the vibrations of C=O and N-H, suggesting that the secondary structure of BSA has been altered during Se@BSA NPs synthesis. FTIR spectra were obtained for free Chi, Se@BSA, and Se@BSA@Chi in order to verify the fabrication of Se@BSA@Chi NPs. In the spectrum of Chi, peaks at 2928, 2958, and 1647 cm^−1^ corresponded to the stretching vibrations of C−H, C−C, and C−N groups, respectively. In the FTIR spectrum of Se@BSA@Chi NPs, the sharpening of the peak in the region of 1635 cm^–1^ compared to Se@BSA NPs shows the successful coating of chitosan on Se@BSA NPs. Chi also has a peak at 3291–3361 cm^−1^, which are related to N-H and O-H bonds, respectively, and these peaks are present in the diagram of Se@BSA@Chi NPs, which indicates the correct synthesis of this nanostructure.

FTIR spectrum was also used to confirm the binding of MTX to Chi. The FTIR spectrum of methotrexate characterized by peaks at 1657 cm^−1^ (corresponding to the carbonyl of MTX carboxyl acid groups), 3100–13500 cm^−1^ (corresponding to acidic O-H and overlapping amine groups) and 2930 cm^−1^ (related to C-H stretching). FTIR spectrum of Se@BSA@Chi-MTX NPs showed all characteristic peaks of Se@BSA@Chi NPs and a slight shift in the 2930 and 1657 cm^−1^ peaks were also observed. It can be concluded that the MTX successfully conjugated in surface of the Se@BSA@Chi NPs and Se@BSA@Chi-MTX NPs fabricated.

To confirm the successful encapsulation of ODNs (SCR & DEC) inside chitosan, the FTIR spectra of free SCR and free DEC compared with all formulations related to the ODNs. ODNs were characterized by FTIR spectra at 1400 cm^−1^(C-H bending), 1600 cm^−1^ (C=O stretch), and 3440 cm^−1^ (N-H bond), and all these spectra were observed in the FTIR spectrum of the Se@BSA@Chi-SCR NPs, Se@BSA@Chi-SCR-MTX NPs, Se@BSA@Chi-DEC NPs, and Se@BSA@Chi-DEC-MTX NPs with some shifts and changes.

It can be concluded that SCR and DEC were successfully loaded in the Se@BSA@Chi NPs and final formulations (Se@BSA@Chi-SCR-MTX NPs & Se@BSA@Chi-DEC-MTX NPs) were prepared.

### UV-vis results showed correct synthesis of different formulations of nanostructures

UV-vis spectroscopy is usually used to investigate the correct formation of the complex and the structural changes that have occurred in its components. Chemical reduction of selenium ions to SeNPs and the synthesis of all nanostructures were evaluated by UV–Vis spectroscopy. Fig. 3B presents the UV-vis spectrum of SeNPs that shows an absorption peak at approximately 270 nm wavelength for SeNPs, while the modification of the surface of selenium nanoparticles with BSA and Chi have a small change in the peaks as well as the presence of absorption peaks in the region of 270 nm. On the other hand, the absorption peak related to methotrexate (387 nm) was observed in Se@BSA@Chi-DEC-MTX NPs and Se@BSA@Chi-SCR-MTX NPs, which indicates the presence of MTX in the nanoparticle structure.

### DLS and zeta potential analysis showed nano-metric size and negative charge of final formulation

**Figure 4:**
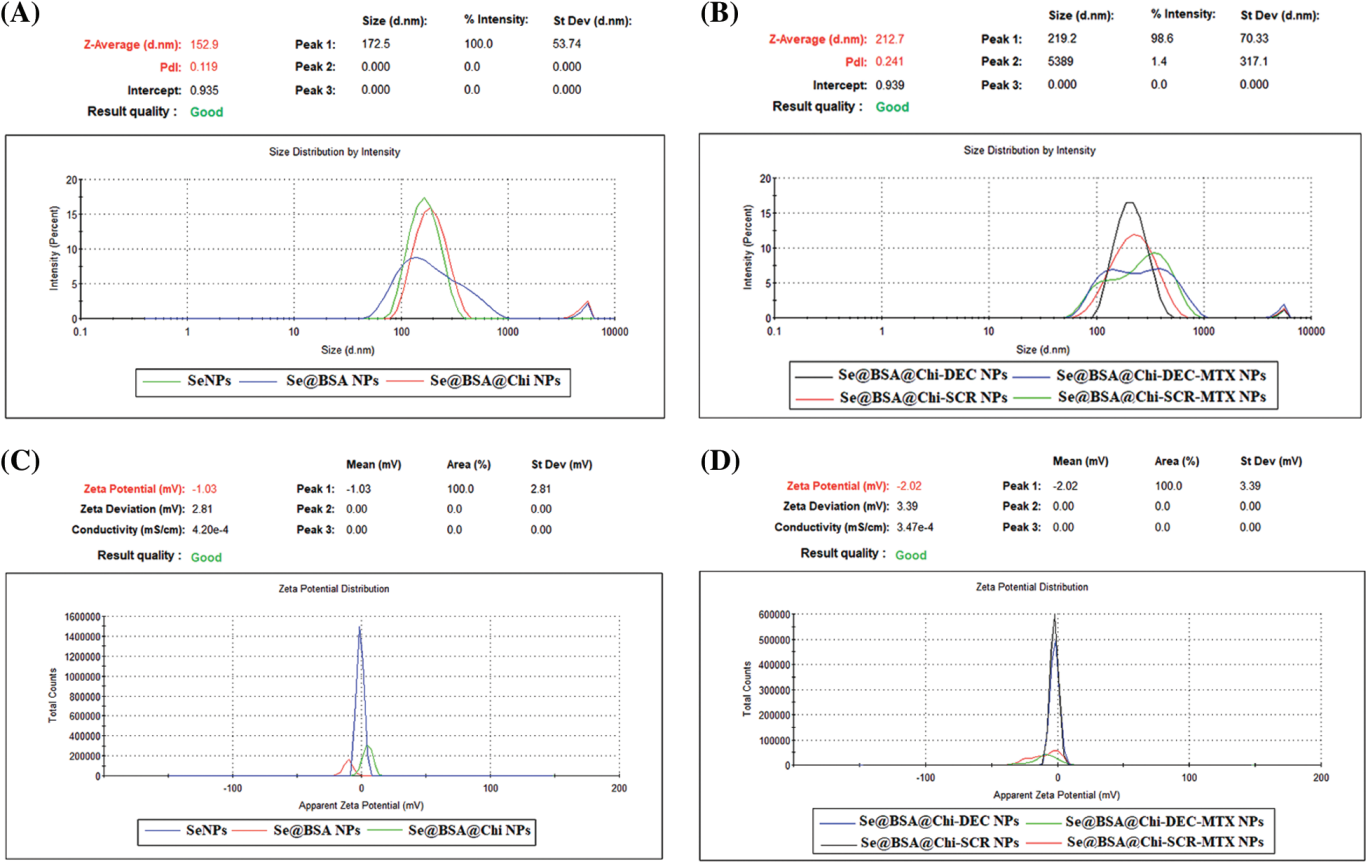
(A and B) DLS particle size analysis showed the 100 to 230 nm size for the different formulations of synthesized NPs and (C and D) Zeta potential distribution of NPs was −0.8 to −9.2 mV based on the materials used in the nanostructures.

With respect to particle size analysis, all synthesized structures were nano-metric (mean diameter <230 nm) and exhibited a relatively narrow size distribution (0.12 < PDI < 0.42). As the structure of the nanoparticles become more complex, the amount of PDI has also increased (Figs. 4A and 4B). Different formulations exhibited distinct charges with z-potential values ranging from −0.8 ± 0.17 to −9.2 ± 0.27 mV. As shown in Figs. 4C and 4D, the averages zeta potential of SeNPs, Se@BSA NPs, Se@BSA@Chi NPs, Se@BSA@Chi-DEC NPs, Se@BSA@Chi-SCR NPs, Se@BSA@Chi-DEC-Chi-MTX NPs, Se@BSA@Chi-SCR-MTX NPs are −0.85, −9.56, +3.75, −2.43, −2.26, −9.27 and −8.8 ± 0.36 mV, respectively (Suppl. Table S1).

### EDX assay confirmed the composition of the nanoparticles through elemental mapping

**Figure S2:**
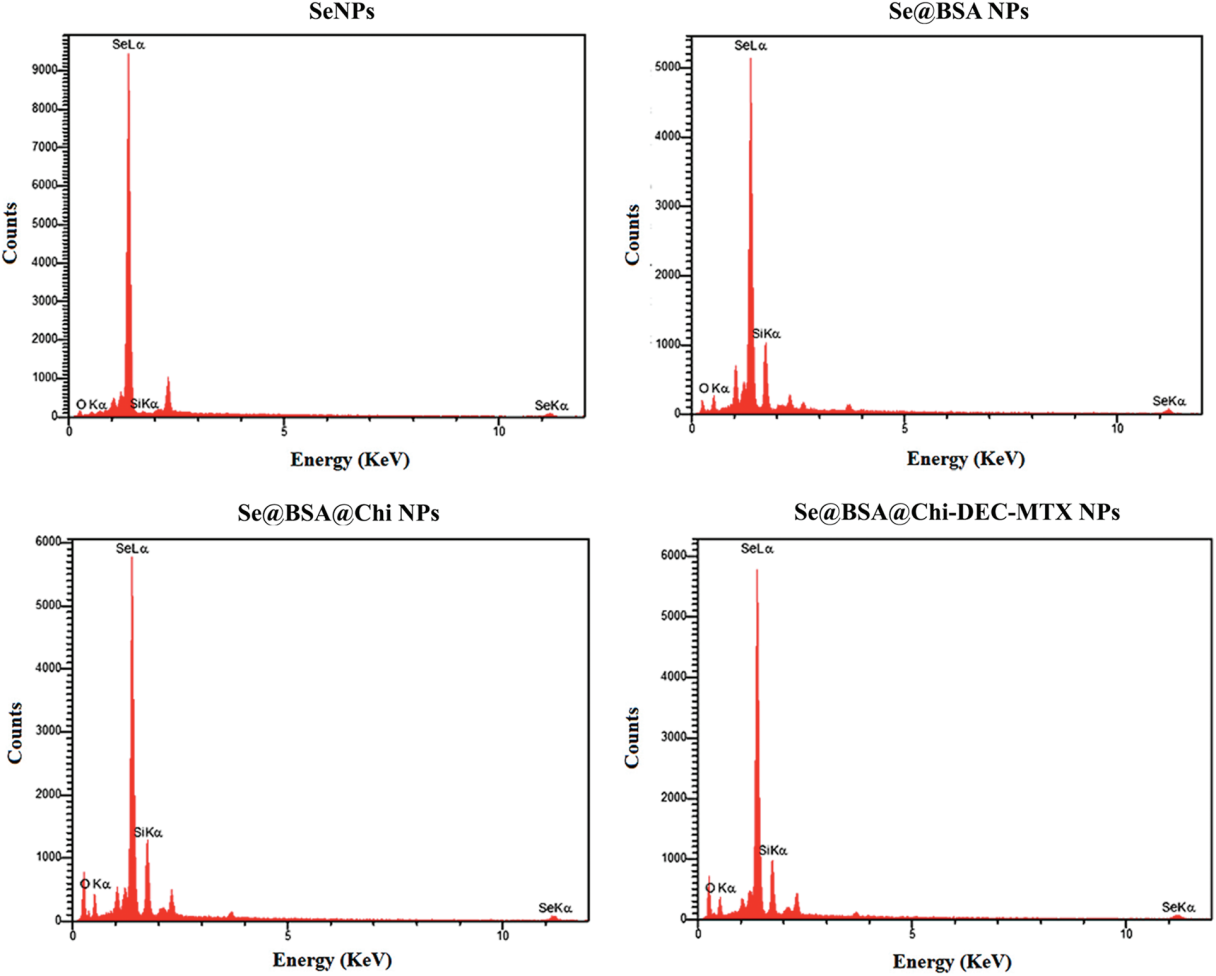
Analysis of different formulations of Selenium nanostructure EDX with its two sharp peaks (SeLα and SeLβ).

The results of energy dispersive X-ray spectroscopy (EDX) showed that SeNPs exhibited a strong signal from the selenium atom (96.11%). Se@BSA NPs, Se@BSA@Chi NPs and Se@BSA@Chi-DEC-MTX NPs also showed a strong signal of Se atoms (72.54%), (67.28%) and (63.21%), respectively (Suppl. Fig. S2).

### TEM analysis showed spherical shape and size of nanoparticles

**Figure 5:**
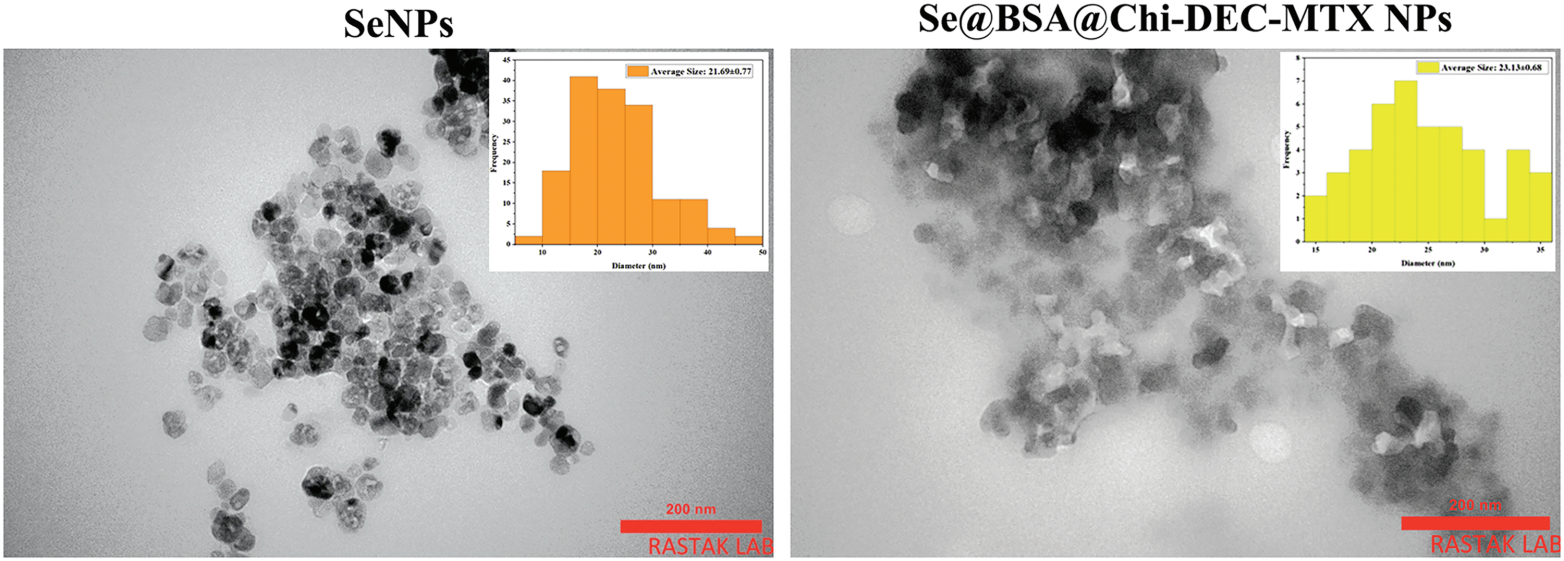
TEM images of SeNPs and Se@BSA@Chi-DEC-MTX NPs showed an average size of 40 nm and a spherical shape.

TEM microphotographs obtained from the SeNPs and the Se@BSA@Chi-DEC-MTX NPs are presented in Fig. 5. The results showed the spherical shape of these nanoparticles as well as their homogeneous particle size distribution with an average size of 40 nm. In addition, an amorphous coating layer was observed on the surface of mineralized NP cores, indicating that the ODNs, Chi and MTX were probably incorporated into SeNPs, which was consistent with the result of FTIR.

### High release of ODNs was performed in acidic environment

**Figure 6:**
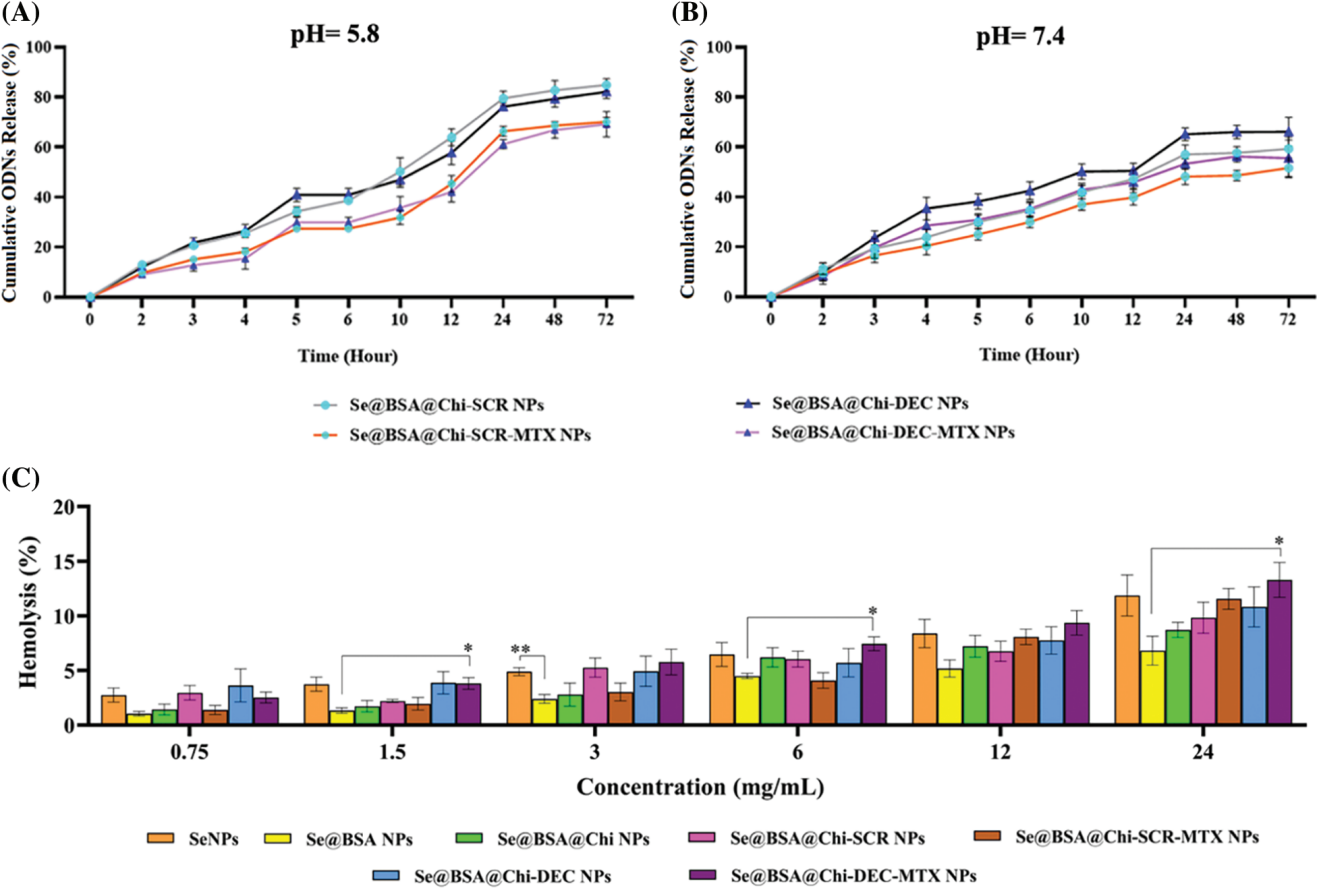
(A and B) Cumulative *in vitro* release of ODNs from NPs. NPs containing ODNs were diluted in PBS at different pH (5.8 and 7.4), incubated at 37°C and shaken horizontally. At preselected time intervals, the released ODNs were separated by ultracentrifugation and the amount of it was measured by spectrophotometer. Each point represents the Mean ± SD obtained from triplicates samples. (C) Hemolysis percentage of human red blood cells in different concentrations of SeNPs, Se@BSA NPs, Se@BSA@Chi NPs, Se@BSA@Chi-SCR NPs, Se@BSA@Chi-DEC NPs, Se@BSA@Chi-SCR-MTX NPs, Se@BSA@Chi-DEC-MTX NPs. *p < 0.05, **p < 0.01.

DEC and SCR release from Se@BSA@Chi-SCR NPs, Se@BSA@Chi-DEC NPs, Se@BSA@Chi-SCR-MTX NPs, Se@BSA@Chi-DEC-MTX NPs in different conditions such as physiological and acidic pH was studied. Each measurement was repeated three times and the average of these three data was used to calculate the released drug concentration. The measurement results were plotted as cumulative released DEC/SCR amount (Fig. 6). The results of examining the release of DEC and SCR attached to the nanoparticle at pH 5.8 show that the amount of ODNs released from Se@BSA@Chi-SCR NPs and Se@BSA@Chi-DEC NPs is approximately 80% and from Se@BSA@Chi-SCR-MTX NPs and Se@BSA@Chi-DEC-MTX NPs is 65% after 72 h (Fig. 6A).

The results of the release study at pH 7.4 were different, so the release rate of ODNs from Se@BSA@Chi-SCR NPs and Se@BSA@Chi-DEC NPs was almost 65% and from Se@BSA@Chi-SCR-MTX NPs and Se@BSA@Chi-DEC-MTX NPs was approximately 50% after 72 h (Fig. 6B).

The release behavior of ODNs from the NPs exhibited a slower and continuous release. Table 1 presents the release rate of ODNs from different formulations varies depending on the pH. The release rate was significantly higher in acidic pH than physiological pH.

**Table 1:** Release rate of ODNs from different formulations in acidic and physiological pH

Sample	Se@BSA@Chi-SCR NPs		Se@BSA@Chi-SCR-MTX NPs		Se@BSA@Chi-DEC NPs		Se@BSA@Chi-DEC-MTX NPs	
pH	5.8	7.4	5.8	7.4	5.8	7.4	5.8	7.4
ODNs release after 2 h (%)	12.96 ± 0.98	11.31 ± 2.34	9.69 ± 0.25	9.42 ± 2.68	12.05 ± 0.44	10.01 ± 3.65	12.74 ± 2.41	8.37 ± 3.35
Cumulative ODNs release after 24 h (%)	79.49 ± 2.84	57.06 ± 3.78	66.31 ± 2.01	48.16 ± 3.19	76.13 ± 1.55	65.11 ± 2.54	61.16 ± 1.84	53.27 ± 2.08
Maximum ODNs depletion (%)	84.84 ± 2.46	59.26 ± 4.69	70.10 ± 1.74	51.57 ± 3.96	82.11 ± 2.67	66.13 ± 5.81	69.14 ± 5.10	55.53 ± 7.31

### Different structures of selenium nanostructures have acceptable hemocompatibility

The blood biocompatibility analysis of prepared nanoparticles was performed based on the hemolysis percentage of human red blood cells of these nanoparticles in different concentrations (0.75, 1.5, 3, 6, 12 and 24 mg/mL) and compared to each other (Fig. 6C). The results show that the observed hemolysis percentage of SeNPs, Se@BSA NPs, Se@BSA@Chi NPs, Se@BSA@Chi-SCR NPs, Se@BSA@Chi-DEC NPs, Se@BSA@Chi-SCR-MTX NPs, Se@BSA@Chi-DEC-MTX NPs were in the range of 2.74% ± 0.6% to 11.86% ± 1.8%, 1% ± 0.2% to 6.8% ± 1.3%, 1.43% ± 0.4% to 8.7% ± 0.68%, 2.9% ± 0.65% to 9.8% ± 1.4%, 1.38% ± 0.4% to 11.55% ± 0.94%, 3.6% ± 1.5% to 10.82% ± 1.83% and 2.59% ± 0.49% to 13.28% ± 1.6%, respectively.

### Cy3-labeled ODNs -loaded NPs accumulate in LNCaP cells

**Figure 7:**
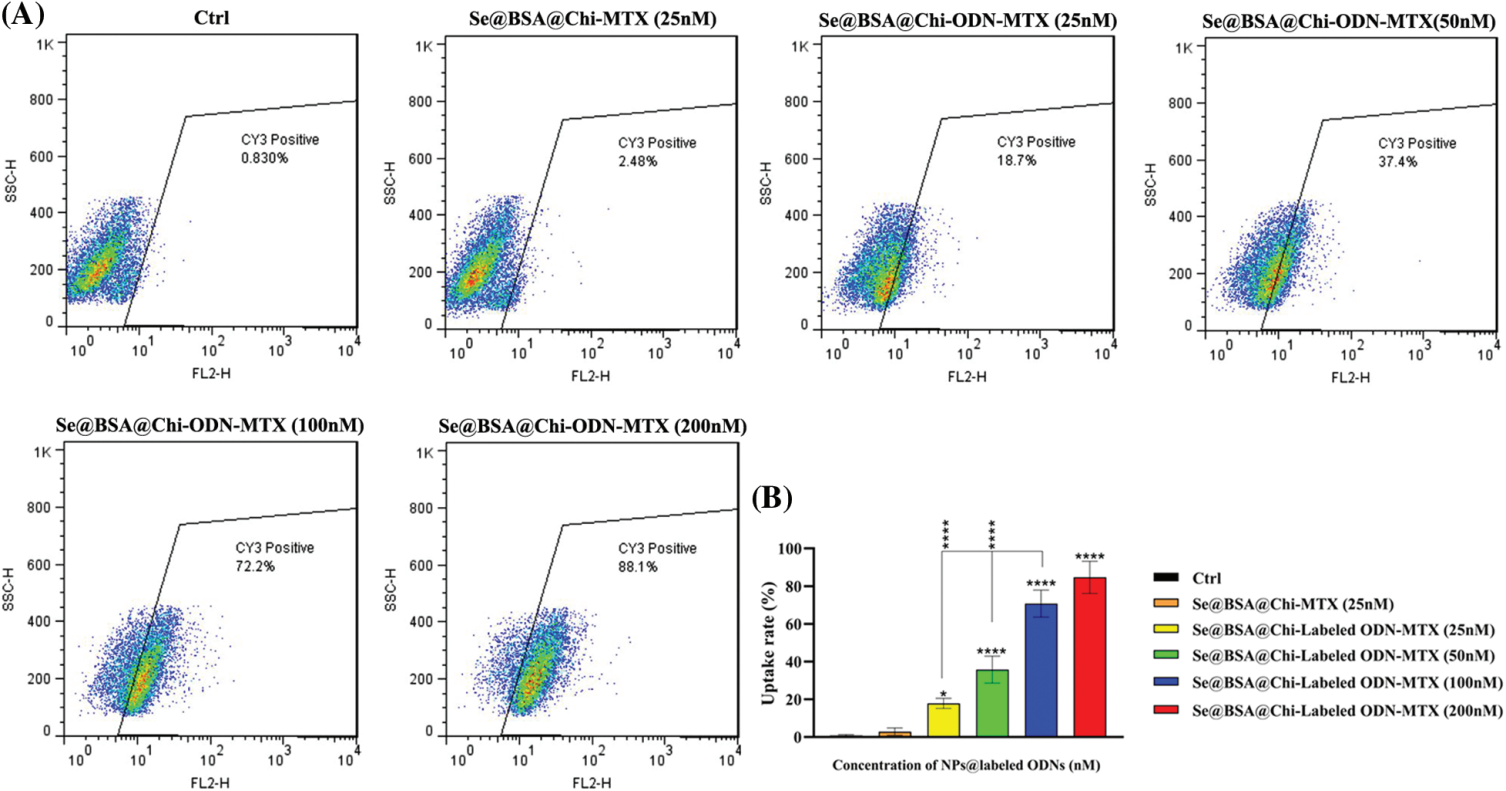
(A) Cell uptake rate of various concentrations of Se@BSA@Chi-labeled ODNs-MTX NPs in LNCaP cells. (B) The column graph shows the uptake rate in transfected cells which detected by flow cytometry. Values with *p < 0.05 and ****p < 0.0001 were regarded as statistically meaningful.

One of the key cellular processes that lead to the internalization of NPs into cancer cells is endocytosis [54]. Uptake results showed that NPs loaded with Cy3-labeled ODNs were transfected and accumulated in the treated LNCaP cells. As shown in Fig. 7, at 24 h post-treatment, the uptake rate of Se@BSA@Chi-labeled ODNs-MTX NPs by LNCaP cells was dramatically increased in a dose-dependent manner to approximately 88% (with increasing the concentration of Labeled-ODNs from 25 to 200 nm) compared to the control (untreated) group and Se@BSA@Chi-MTX NPs treated group (negative control).

### ODNs-loaded NPs reduce cell viability in with and without X-irradiation exposure condition

**Figure 8:**
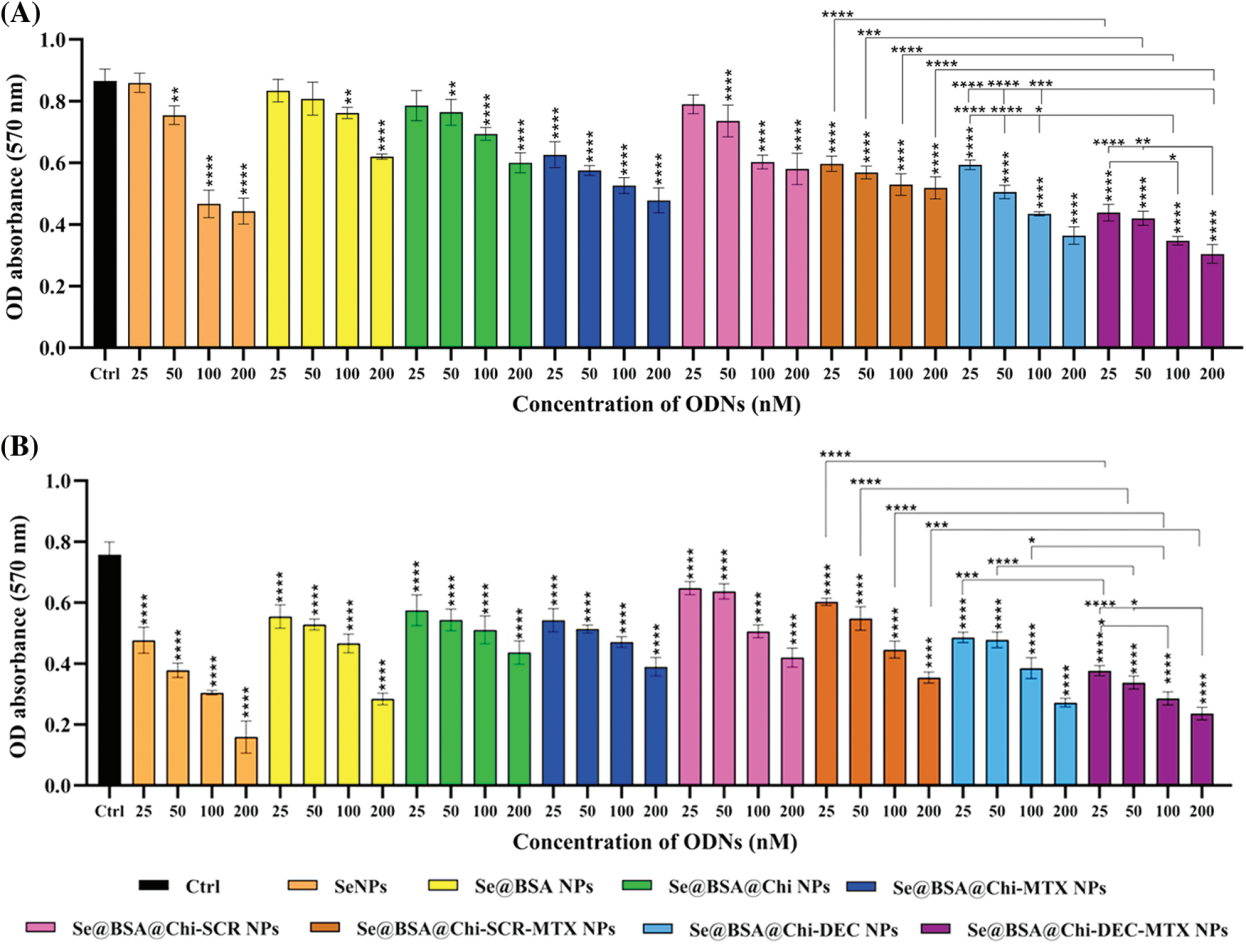
Cell viability evaluation on treated LNCaP cells with various NP groups at 24 h after treatment performed by MTT assay in condition of (A) no X-irradiation and (B) under X-irradiation exposure. Values with *p < 0.05, **p < 0.01, ***p < 0.001 and ****p < 0.0001 were regarded as statistically meaningful.

The cytotoxic effects of different groups of nanostructures (25 to 200 nM) on LNCaP cancer cells in both no X-irradiation (Fig. 8A) and under X-irradiation (Fig. 8B) conditions were evaluated by MTT assay. Without X-irradiation exposure, all groups of NPs compared with the control (untreated) group showed cell viability decrease in a dose-dependent manner 24 h after treatment of cells. The maximum decrease in cell viability was observed when LNCaP cells were incubated with 200 nM Se@BSA@Chi-DEC-MTX NPs. However, this reduction rate by all investigated concentrations of Se@BSA@Chi-DEC-MTX NPs was significantly higher than the control group and other nanoparticle formulations. In treated groups by SeNPs (25 nM) and Se@BSA NPs (25 and 50 nM) no toxicity was observed in comparison with control group. The decrease in cytotoxicity rate in Se@BSA NPs compared to SeNPs could be due to BSA coating on SeNPs. In addition, there was significant cytotoxicity difference for Se@BSA@Chi-DEC NPs and Se@BSA@Chi-DEC-MTX NPs in compared with Se@BSA@Chi-SCR NPs and Se@BSA@Chi-SCR-MTX NPs under the same conditions, which indicates the specific function of Myc decoy ODNs on reducing proliferation and cell viability. In the NP groups that conjugated with MTX, cell viability was significantly reduced compared to other groups that indicated the effect of MTX in targeting and cytotoxicity on LNCaP cells (Fig. 8A).

Treatment of cells at 25–200 nM concentrations of all NP groups in comparison with control group exhibited significant toxicity against LNCaP cells under X-irradiation conditions. A comparison of the results obtained from both with and without X-irradiation exposure conditions, shows a significant cell viability reduction when cells were exposed to X-irradiation, especially in treatment with NPs containing DEC ODNs. So the anti-cancer effect of Se@BSA@Chi-DEC-MTX NPs on LNCaP prostate cancer cells acts by blocking Myc transcription factor intensifies during X-irradiation exposure (Fig. 8B). According to the obtained results of MTT assay, the best IC_50_ value was 100 nM so we used this concentration to do other tests.

### ODNs-loaded NPs induce cell cycle arrest at G0/G1 phase in no X-irradiation exposure condition

As shown in Suppl. Fig. S2, at 24 h after treatment with different formulations and the same concentration (100 nM) of nanostructures (Se@BSA@Chi NPs, Se@BSA@Chi-MTX NPs, Se@BSA@Chi-SCR NPs, Se@BSA@Chi-DEC NPs, Se@BSA@Chi-SCR-MTX NPs, Se@BSA@Chi-DEC-MTX NPs), in without X-irradiation condition, cell populations at the S and G2/M phases were clearly reduced, and meanwhile, the highest amounts of cell cycle arrest at G0/G1 phase were occurred after the cells were treated with the Se@BSA@Chi-DEC NPs and Se@BSA@Chi-DEC-MTX NPs in compared with control (untreated) group. Nevertheless, the G0/G1 arrest rate was very low in cells treated with Se@BSA@Chi-MTX NPs.

Se@BSA@Chi NPs and Se@BSA@Chi-SCR nanoparticle-treated groups as compared with the control group revealed an insignificant difference in the extent of the G0/G1 phase. This result could indicate that SCR ODNs have no role in cell cycle arrest. Our results revealed that the arrest at G0/G1 phase in the treated cell group with Se@BSA@Chi-DEC-MTX NPs was more than in the other groups that could be indicate the synergistic effects of MTX and Myc decoy ODNs on increasing LNCaP cancer cells suppression.

**Figure S3:**
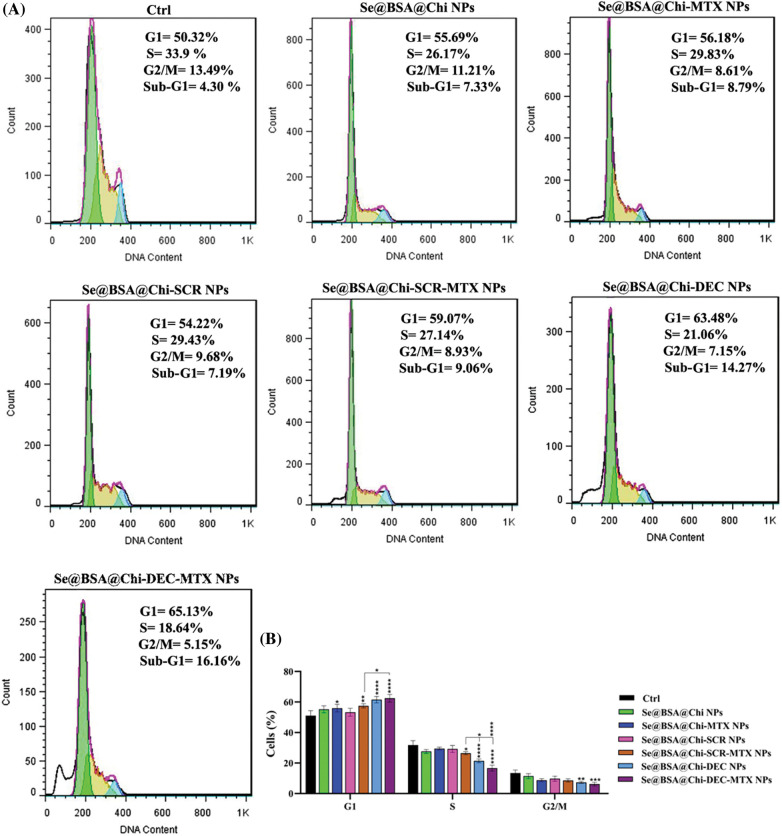
(A) Evaluation of different NPs effects with the same concentration (100 nM) on the cell cycle of LNCaP cells at 24 h post-treatments and without X-irradiation condition. (B) The column graph shows the cell cycle arrest rate in treated cells which detected by flow cytometry. Two-way ANOVA was used for analysis. *p < 0.05, **p < 0.01, ***p < 0.001, ****p < 0.0001.

In addition, there was a significant arrest in the treated cells with Se@BSA@Chi-DEC-MTX NPs in comparison with Se@BSA@Chi-SCR-MTX NPs under the same conditions in G0/G1 phase. The present results indicated that the knockdown of Myc might inhibit the proliferation of LNCaP cells by inducing the cell cycle arrest at the G0/G1 phase. The amount of cell population in the G0/G1 phase in the cell group treated with Se@BSA-Chi-MTX NPs was significantly higher than in the Se@BSA@Chi NPs group, which can indicate the effect of the MTX in the formulation of the Se@BSA@Chi-MTX group (Suppl. Fig. S3).

### ODNs-loaded NPs induce cell cycle arrest at G2/M phase in X-irradiation exposure condition

**Figure 9:**
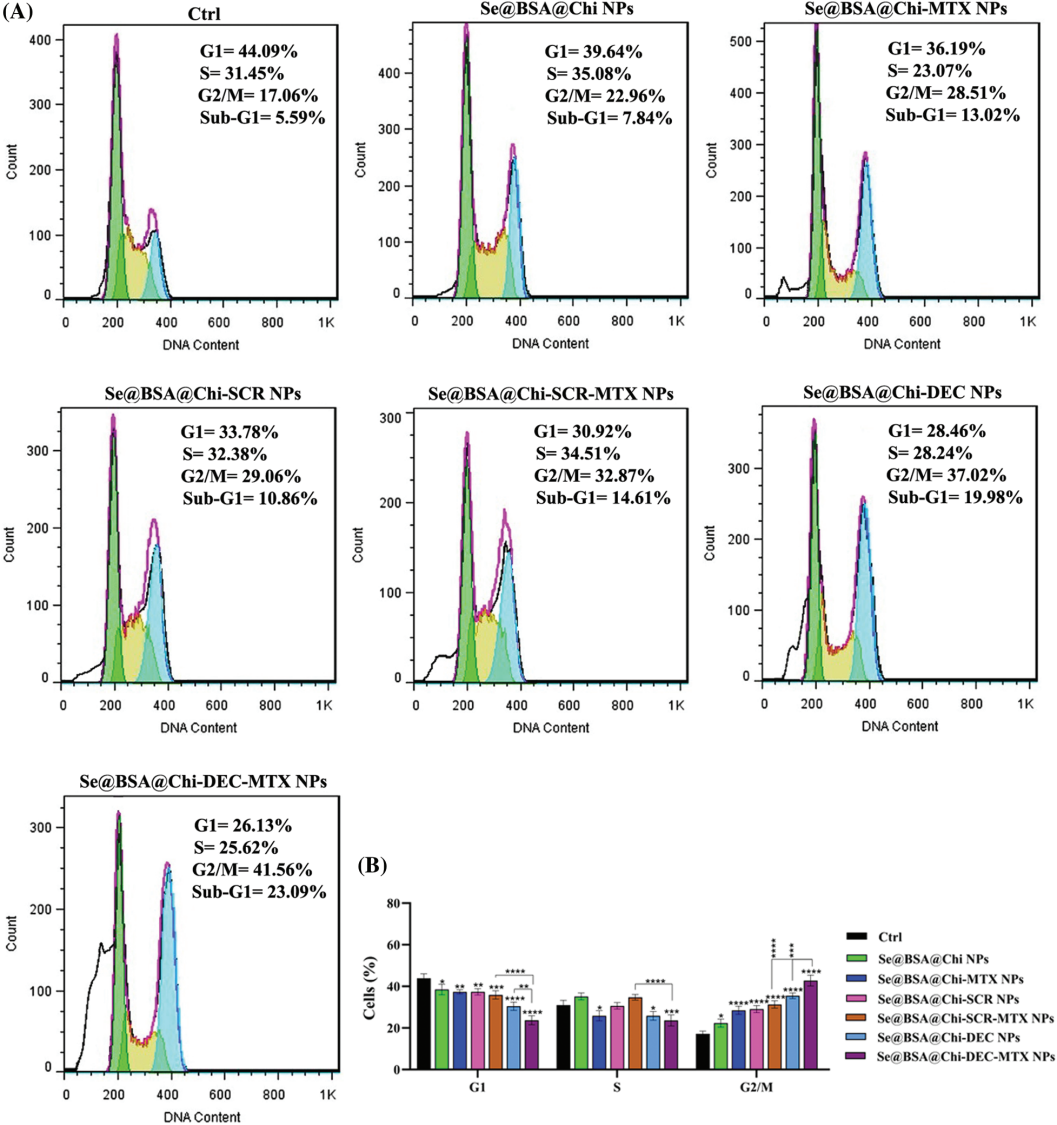
(A) Evaluation of different NPs effects with the same concentration (100 nM) on the cell cycle of LNCaP cells at 24 post-treatments and in X-irradiation condition. (B) The column graph shows the cell cycle arrest rate in treated cells which detected by flow cytometry. Two-way ANOVA was used for analysis. *p < 0.05, **p < 0.01, ***p < 0.001, ****p < 0.0001.

Combinational treatment on LNCaP cells with 100 nM of different formulations of nanostructures (Se@BSA@Chi NPs, Se@BSA@Chi-MTX NPs, Se@BSA@Chi-SCR NPs, Se@BSA@Chi-DEC NPs, Se@BSA@Chi-SCR-MTX NPs, Se@BSA@Chi-DEC-MTX NPs) and X-irradiation significantly induced an increase of cell population in the G2/M phase and a decrease of cells in the G0/G1 phase as compared with the control (untreated) group cells (Fig. 9).

Se@BSA@Chi-DEC-MTX NPs significantly arrested cell population at G2/M, the radiosensitive phases of the cell cycle, more than other NPs-treated groups that indicating effects of Myc decoy and SeNPs in increasing sensitivity of LNCaP cells to the X-irradiation. The amount of cell arrested in G2/M phase in the cell group treated with Se@BSA@Chi-MTX NPs was significantly higher than treated group with Se@BSA@Chi NPs, which indicates the effect of the MTX in the formulation that can have synergetic effect in combination with X-irradiation (Fig. 9).

### ODNs-loaded NPs increase apoptosis rate in no X-irradiation exposure condition

As shown in Suppl. Fig. S3, the total apoptosis (early and late) rate in 24 h post-treatment with all NPs-treated groups (Se@BSA@Chi NPs, Se@BSA@Chi-MTX NPs, Se@BSA@Chi-SCR NPs, Se@BSA@Chi-DEC NPs, Se@BSA@Chi-SCR-MTX NPs, Se@BSA@Chi-DEC-MTX NPs) at a concentration of 100 nM significantly increased as compared to the control (untreated) group.

The apoptosis rate in the cell group treated with Se@BSA@Chi NPs in comparison with Se@BSA@Chi-SCR NPs treated group was low and insignificant. This result may indicate that SCR ODNs have no role in cellular apoptosis induction. Also, treated groups with Se@BSA@Chi-MTX NPs and Se@BSA@Chi-SCR-MTX NPs showed similar effects on apoptosis rate indicating the effect of methotrexate alone and SCR has no effect.

**Figure S4:**
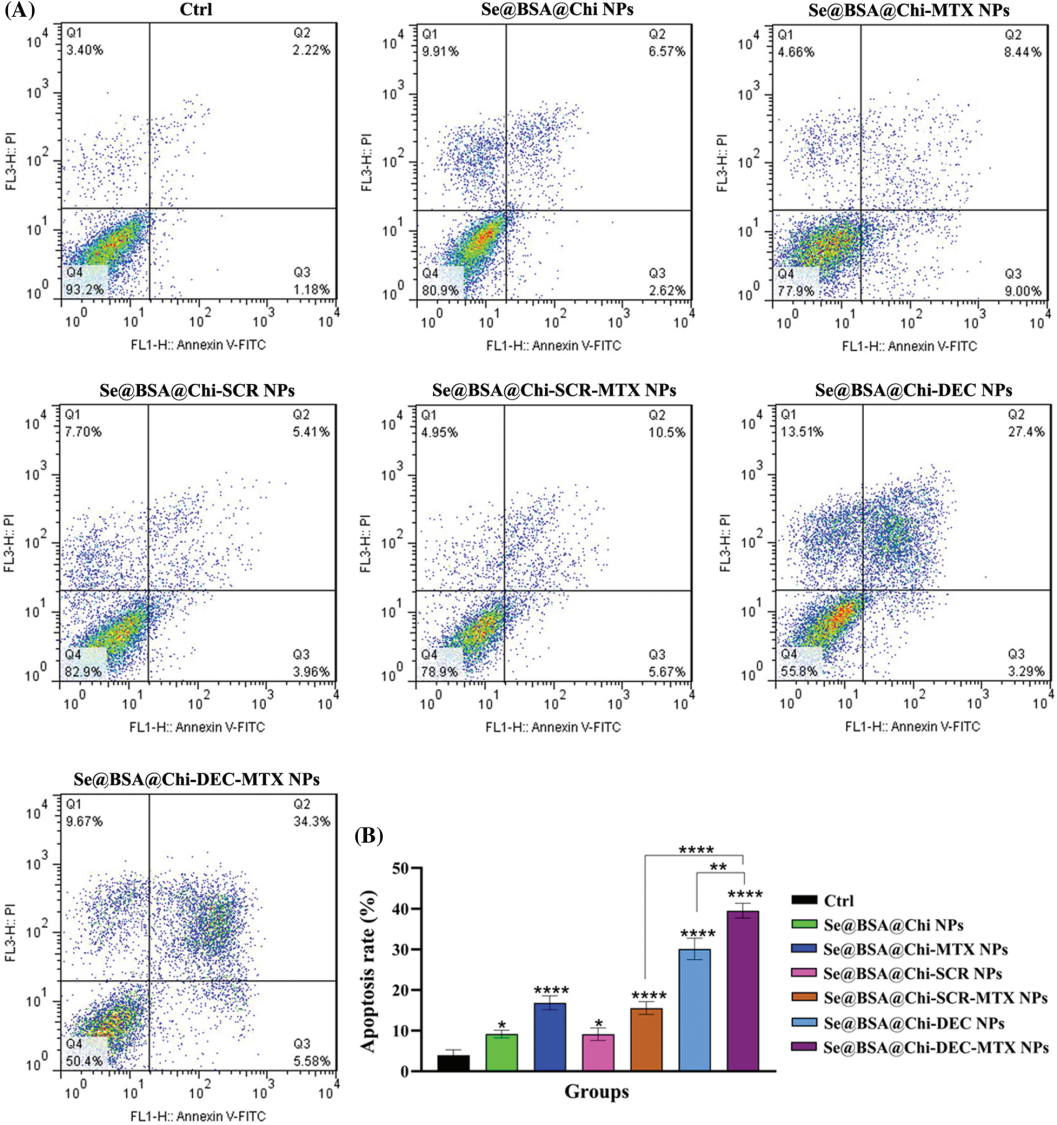
(A) Evaluation of different NPs effects with the same concentration (100 nM) on the apoptosis rate of LNCaP cells at 24 h post-treatments and in without X-irradiation exposure condition. (B) The column graph shows the apoptosis rate in treated cells which detected by flow cytometry. One-way ANOVA was used for analysis. *p < 0.05, **p < 0.01, ****p < 0.0001.

The total apoptosis rate (early: 5.58% and late: 34.39%) in the cell group treated with Se@BSA@Chi-DEC-MTX NPs was significant in compared to the other groups. It suggests the synergistic effect of Myc decoys and MTX drug on induce apoptosis of LNCaP cells (Suppl. Fig. S4).

### ODNs-loaded NPs increase apoptosis rate in X-irradiation exposure condition

**Figure 10:**
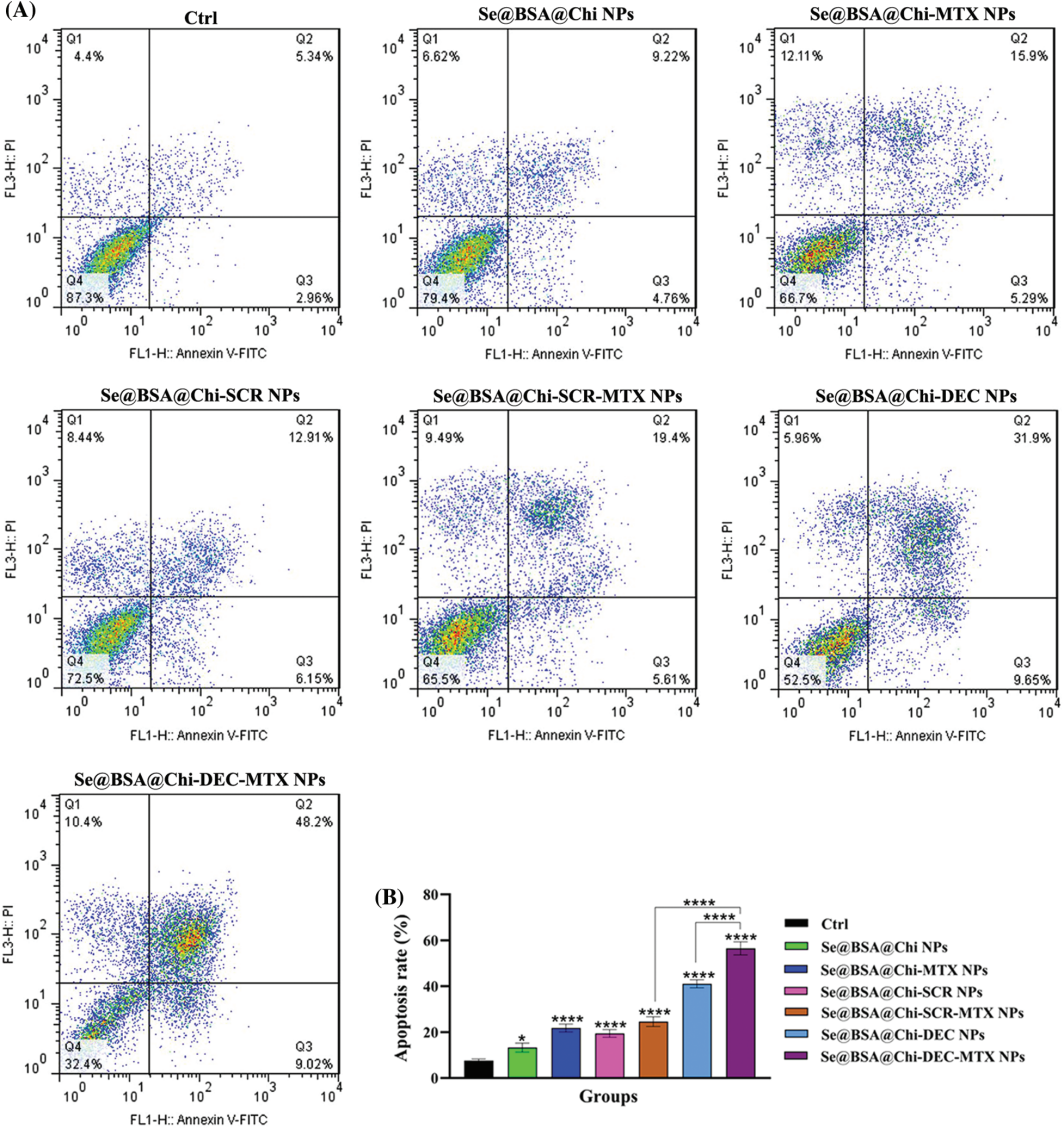
(A) Evaluation of different NPs effects with the same concentration (100 nM) on the apoptosis rate of LNCaP cells at 24 h post-treatments and in X-irradiation exposure condition. (B) The column graph shows the apoptosis rate in treated cells which detected by flow cytometry. One-way ANOVA was used for analysis. *p < 0.05 and ****p < 0.0001.

Obtained result showed that all nanostructure formulations combined with X-irradiation induce apoptosis in treated cells. Apoptosis rate in the Se@BSA@Chi-DEC-MTX NPs group under X-irradiation (early: 9.02% and late: 48.02%) was significantly high compared to the other groups. In addition, Se@BSA@Chi NPs (early: 4.76% and late: 9.22%) had a less significant effect on the apoptosis of LNCaP cells compared with other groups (Fig. 10).

The effect of Se@BSA@Chi-DEC-MTX NPs and Se@BSA@Chi-DEC NPs on cell apoptosis compared to the Se@BSA@Chi-SCR-MTX NPs and Se@BSA@Chi-SCR NPs was significantly high that indicating Myc-specific function and synergetic effects of Myc decoy ODNs and MTX drug on radiation therapy. The rate of apoptosis in cells treated with Se@BSA@Chi-SCR-MTX NPs and Se@BSA-@Chi-MTX NPs is approximately the same, indicating that SCR does not affect.

As shown in Fig. 10, compared to the control group, cells treated with Se@BSA@Chi NPs, Se@BSA@Chi-MTX NPs, Se@BSA@Chi-DEC NPs, Se@BSA@Chi-SCR NPs, Se@BSA@Chi-DEC-MTX NPs and Se@BSA@Chi-SCR-MTX NPs in combination with X-irradiation can induce cellular apoptosis more than without X-ray irradiation exposure conditions.

### ODNs-loaded NPs inhibit cell migration in with and without X-ray irradiation exposure condition

**Figure 11:**
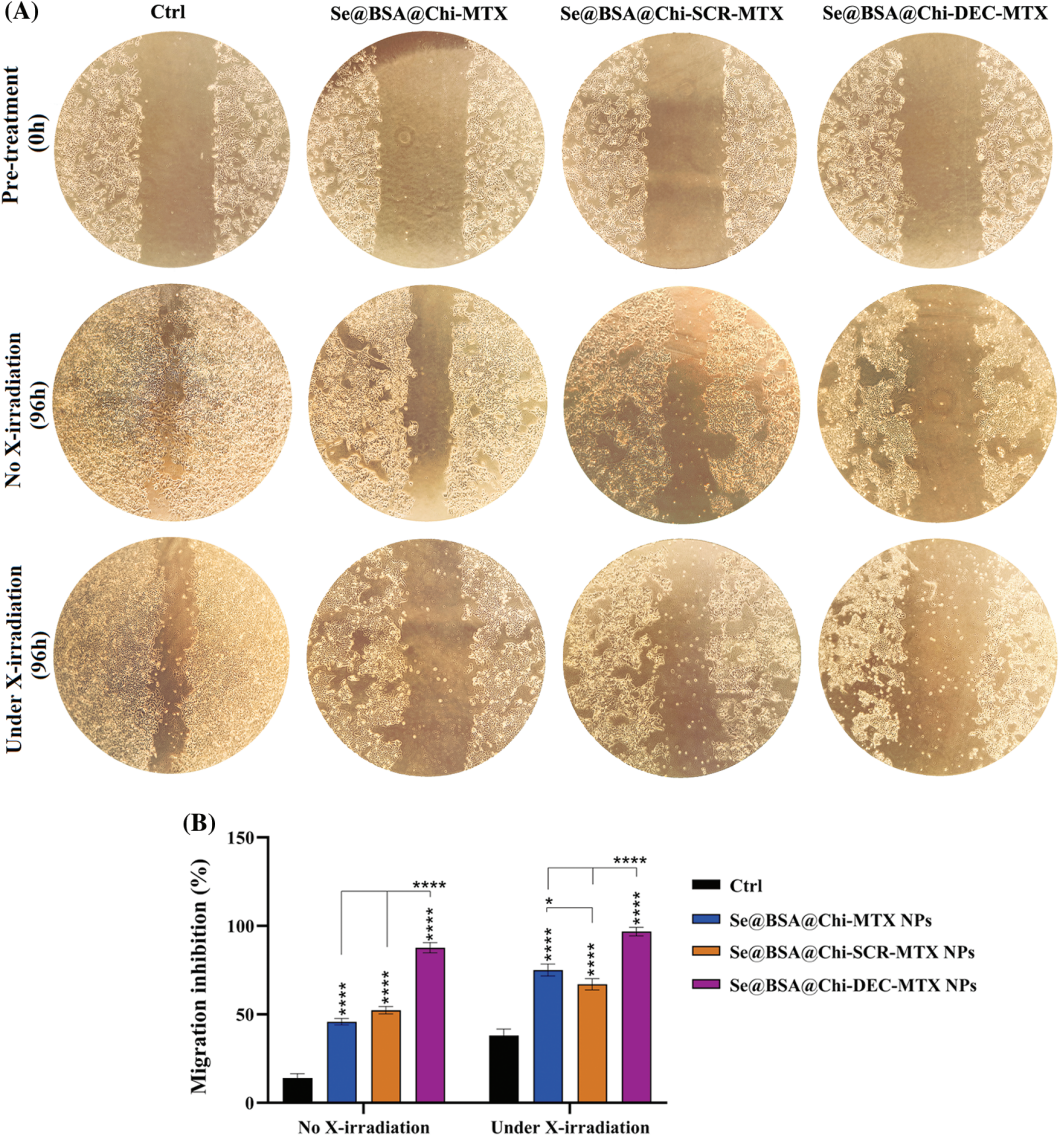
(A) Inhibition evaluation of cell migration by a scratch assay in LNCaP cells by 96h post-treatment with 100 nM of Se@BSA@Chi-MTX NPs, Se@BSA@Chi-SCR-MTX NPs, and Se@BSA@Chi-DEC-MTX NPs under without and with X-irradiation condition. (B) The column graph shows the cell migration inhibition rate in treated cells. Two-way ANOVA was used for analysis. *p < 0.05 and ****p < 0.0001.

Our results showed that under no X-ray irradiation conditions, Se@BSA@Chi-MTX NPs, Se@BSA@Chi-SCR-MTX NPs, and Se@BSA@Chi-DEC-MTX NPs effectively suppressed the migration of LNCaP cells in compared to control (untreated) group (Fig. 11).

The highest rate of cell migration inhibition is related to Se@BSA@Chi-DEC-MTX NPs which indicates the specific action of the Myc decoy oligodeoxynucleotide and MTX drug. Inhibition of cell migration in groups treated with Se@BSA@Chi-MTX NPs and Se@BSA@Chi-SCR-MTX was approximately equal, indicating that SCR had no significant effect on cell migration.

Under the X-irradiation conditions, the rate of cell migration inhibition in all NPs-treated groups was significantly high compared with the control group. Induction of migration inhibition by Se@BSA@Chi-DEC-MTX NPs treatment was higher than in the other treated groups. This result can also show the synergistic effect of Myc decoy ODNs, MTX drug, and selenium nanoparticles presented in the final formulation of NPs on radiation therapy in cell migration inhibition (Fig. 11).

## Discussion

Conventional treatments for prostate cancer, especially chemotherapy, have faced many challenges such as low drug accumulation at the cancer site, rapid clearance of the drug from the body, and drug resistance. Small size, high surface area, high stability and easy adaptability have made nanomaterials suitable carriers of chemotherapy drugs and as well as suitable agents for phototherapy, thermal therapy and radiation sensitizers [55]. Among the nanomaterials designed and synthesized for therapeutic applications, radiation-sensitizer nanostructures have been considered recently [56]. So that, nanosystems with the ability to carry drugs in addition to radiation sensitivity have received more attention [57].

It has been reported that combination of SeNPs with regular anticancer reagents increases the efficacy of chemotherapeutic reagents and reduces adverse outcomes [58]. Synthesizing of SeNPs is desired via a biological route to improve the stability and biocompatibility of compounds [59]. Most of the malignancies show multi-drug resistance to drugs, and these drugs have systemic toxicity, which may be solved by reducing the dosage of the drug. Several drugs have been used for this purpose in combination with SeNPs or as complexes/conjugates with SeNPs [60]. Several nanoparticle-mediated therapeutic nucleic acids are currently at different stages of preclinical studies, and some are in clinical trials [61,62]. In TFD strategy, small oligodeoxynucleotides (ODNs) sequences by binding to the target transcription factors, reduce the probability of their binding to the main promoter sequence on the genome and thus prevent the expression of downstream genes [63]. In this study, a nanostructure was designed and synthesized using selenium nanoparticles modified with BSA, coated with Myc decoy ODNs that encapsulated in chitosan and conjugated with methotrexate (Se@BSA@Chi-DEC-MTX NPs) for the treatment of PC. The efficiency of the prepared nanoparticle was investigated *in vitro*.

In the present investigation, the tertiary structure of the DEC and SCR ODNs sequences was studied computationally. Next, the interaction pattern of the bHLH domain of Myc protein towards ODNs was evaluated by molecular docking. Myc protein is a transcription factor that recognizes the core sequence 5′-CAC[GA]TG-3′ [64]. The preserved binding site in the sequence of decoy ODN indicated several interactions with the bHLH domain of Myc including R356, R357, H359, N360, R364, R366, R367, and K392. Mutations in the sequence of scramble ODN has resulted in relatively different interaction pattern including R356, N360, K371, K389, and K392. The Myc/DEC interactions were consistent with the results of the crystal structure of the Myc/MAX complex bound to DNA that showed the contact of Myc sequence with the DNA nucleotides by residues K355, R356, H359, E363, and R367 in Myc [50]. Finally, the structural stability of the complexes was assessed during 50 ns MD simulations. The Myc/DEC complex displayed not only the minimum structural deviation during the simulation but also fewer fluctuations indicated in the radius of the gyration plot that signified the well-set structure of Myc in the interaction with DEC compared with SCR. Consistent with molecular docking, Myc/DEC complex contained more H-bonds during the simulation that trigger higher binding affinity and stability compared to Myc/SCR. However, the computational findings concerning the higher stability and affinity of the Myc/DEC were evaluated through the following *in vitro* experiments.

The results obtained from nanostructure characterization tests performed for the synthesized nanoparticles indicated the correct synthesis of these nanoparticles. BSA-modified nanoparticles can be temporarily “invisible” in blood circulation, which prolongs the blood circulation time and allows nanoparticles to exert a better therapeutic effect [65]. In this study, the reduction of sodium selenite and its transformation into selenium nanoparticles proved by changing the color of the solution containing nanoparticles from colorless to dark orange. Along with chitosan (Chi), we coated our ODNs (SCR/DEC) on Se@BSA NPs and made Se@BSA@Chi-ODNs NPs.

Chitosan is a positively charged biological soft molecule that can be electrostatically proposed to the surface of Se@BSA NPs to both protect the negatively charged ODNs coated on the nanoparticles and enhance NPs selective uptake and anticancer activity. The positive charge of the NH3+ group on the outer surface of the Se@BSA@Chi NPs contributed to the high stability of these NPs in aqueous solutions. In addition, the electrostatic interactions between the anionic carboxylic groups of MTX molecules and the cationic groups of Chi in the Se@BSA@Chi-ODNs-MTX NPs can cause the binding of this chemical drug to the nanostructure.

In the present study, Chi incorporation affected the z-potential of Se@BSA NPs and changed it from −10 to +3 mV (An increase as much as 13 mV), which could be due to the presence of NH3+ groups of chitosan. Regarding the formulations prepared in the presence of ODNs, drug incorporation affects the z-potential of the nanoparticles and made it negative. Adding methotrexate to the nanostructure made the nanostructure more negative (about −9 mV), which could be due to the presence of anionic carboxyl groups. PDI value was lower than 0.42. According to previous studies, the value of Polydispersity Index less than 0.5 are considered as suitable particle size distribution [66]. The hydrodynamic size (obtained from DLS) of synthesized nanoparticles with different formulations was measured between 100 to 230 nm. In addition, TEM results showed a spherical morphology a size of 40 to 50 nm. Elemental composition analysis showed the presence of strong signals from the Se atom.

FTIR analysis was performed to characterize the surface chemistry of nanoparticles with different formulations. The results obtained by changing the specific peaks of the compounds participating in the synthesis of nanoparticles showed the successful coating and combination of these components in different formulations of nanoparticles which were in line with other similar studies [67,68].

The UV-vis spectrum shows an absorption peak at approximately 270 nm wavelength for SeNPs, which is in line with the results obtained from other studies that obtained a peak at 265 nm for SeNPs [69,70]. The peak related to the spectrum of methotrexate drug was observed in nanoparticles containing this drug. In addition, the spectra of the synthesized nanoparticles were different from each other due to different sizes and coatings of synthesized SeNPs with different compounds.

In this study, the obtained result showed that the release rate of ODNs in acidic pH was higher than in physiological pH. Since the pH of the endosome environment in tumors is acidic, this can be a positive point and a worthy feature for synthetic nanoparticles. Chitosan shows high solubility due to the protonation of its amino groups in acidic environment. In addition, at acidic pH, the electrostatic attraction between protonated amine groups and H_2_O molecules is more significant, causing more dissolution of CS polymers and rapid drug release [71]. By dissolving chitosan, it becomes easier to release ODNs encapsulated in its web on the nanoparticles and loaded on the selenium nanoparticle. Since the environment of cancer cells (Endosome) is acidic, the rate of release of this chemotherapy drug in this environment is high [72], and as it is clear from the results of the tests conducted in this study, this drug together with gene therapy has a synergistic effect on the survival and apoptosis of LNCaP cancer cells.

Materials resulting in more than 5% hemolysis are reported to be hemolytic, between 5% and 2% as mildly hemolytic, and less than 2% as nonhemolytic [73]. The small size NPs, high concentration and longer exposure time of them can cause hemolysis and morphological changes in red blood cells [74]. One of the most common experiments in the study of the interaction of nanoparticles with blood components is to determine their hemolytic properties. Therefore, it is worthwhile to explore the hemocompatibility of selenium NPs before using them in biomedical applications. The quantitative data did not show significant release of hemoglobin in samples treated with low concentrations of different nanoparticle formulations. However, at high concentrations partial hemolysis was observed. The previous studies on hemolysis assay have shown that the hemolysis properties of nanoparticles can be different based on surface coverage, type, size, nature and surface charge [75].

In this study, selenium nanoparticles designed and synthesized to deliver Myc decoy ODNs and methotrexate drug (which was also used as the target ligand) to cancer cells and investigated gene regulation and chemotherapy synergistic effects under X-ray irradiation exposure.

In our study, the obtained results indicated that the cellular absorption of Cy3-labeled Myc decoy loaded on Se@BSA@Chi NPs that conjugated with MTX (Se@BSA@Chi-labeled ODN-MTX NPs) increased with a dose-dependent pattern. Plenty of MTX-loaded NPs have been used to decrease the side effects and increase targeted delivery and medical efficiency of the compounds, and their effectiveness has been reported in several malignancies. As explained in the introduction section, studies have shown that nanoparticles targeted with folic acid enter prostate cancer cells through PSMA [26], and since methotrexate is an analog of folic acid, it can increase the absorption of nanoparticles through this route.

In the present study, the cell viability reduction in cell group treated with nanoparticles containing decoy and methotrexate (Se@BSA@Chi-DEC-MTX NPs) was high compared to other nanoparticle groups. This cell viability reduction was very high in combination with X-irradiation which indicates the synergistic effect of these treatment methods in combination with each other. The viability rate of cells treated with nanoparticles containing Myc decoy ODNs was much lower than the cells treated with nanoparticles containing scramble ODNs, which indicates the specific action of Myc decoy. In addition, the results showed a significant effect of methotrexate in reducing cell viability. As mentioned a study has shown that the release of methotrexate in acidic environment was more due to the protonation of carboxyl groups in the MTX moiety, which are sensitive to external pH changes in weakly acidic conditions [72]. Since the environment of cancer cells is acidic, it is possible that some MTX was released from nanoparticles and affected the survival and apoptosis of LNCaP cells.

Treatment of cells with selenium nanoparticles (SeNPs) decrease cell viability, but this decrease was lower in the Se@BSA NPs-treated group, which can attribute to the decrease in the toxicity of SeNPs due to BSA coating. Earlier studies have claimed that SeNPs show a potent inhibitory effect on cell growth of LNCaP and PC-3 cancer cells *via* suppressing the androgen receptors expression at transcriptional and translational stages [76,77]. This inhibitory effect was due to the endocytosis of SeNPs and the intense production of mitochondrial Reactive oxygen species (ROS) along with the reduction of ATP, which indicates mitochondrial damage caused by SeNPs [78]. Under X-irradiation conditions, the cell viability was reduced further in treatment groups. This result suggests the effective potential of the combination of SeNPs and X-irradiation exposure as a promising approach for cancer combinational therapy. The study showed that MiR-449a was upregulated and c-Myc was downregulated following X-irradiation (IR) in LNCaP cells, so that overexpression of miR-449a or knockdown of c-Myc increased the sensitivity of LNCaP cells to IR [79]. Previous reports have claimed that the photoelectric absorption and secondary electron caused by gamma or X-irradiation could produce ROS. ROS are necessary for regulating the cancer cell fate at radiotherapy and chemotherapy [80,81].

Cell cycle arrest is important for cell proliferation reduction caused by antitumor therapeutic compounds [82]. The obtained result revealed that, under no X-ray irradiation condition the cell cycle arrest occurred at the G1 phase in LNCaP cells after treatment with 100 nM nanostructure. Under X-irradiation conditions, in the NPs-treated groups, the proportion of cells in the G2/M phase significantly elevated, so that these changes were more significant in Se@BSA@Chi-DEC NPs and Se@BSA@Chi-DEC-MTX NPs-treated groups. A study showed that down-regulation of c-Myc expression by siRNA approach caused activation of the CDK inhibitor p21 and in a cell cycle arrest at the G0/G1 phase [83]. Although, the mechanisms and the factors underlying the G2/M cell cycle arrest by nanoparticles are still not clear. A recent study by Mahmoudi et al. reported that the nanoparticles effects on cell cycle could depend on their intracellular location [84]. It is clear that cells indicate various radio sensitivities at different phases of cell cycle. Cells in late S-phase have the most potent radio-resistance and those in the G2/M phase are most sensitive [85,86].

Our results demonstrated that the total apoptosis rate in the cell group treated with Se@BSA@Chi-DEC-MTX NPs was significant in compared to the other groups that could be indicating the synergistic effect of Myc decoys and MTX drug on inducing apoptosis of LNCaP cells. Applying radiotherapy increased the rate of cell apoptosis in cell groups treated with different groups of nanoparticles compared with no X-ray irradiation conditions. SeNPs are generally supposed to be able to trigger tumor cell apoptosis by increasing cellular uptake and cellular ROS levels via the expression of *Bcl-2*/Bax, and activation of caspase-3 [30,87]. Also, a study has shown that the effect of Methotrexate (MTX)-loaded chitosan (CS) nanoparticles on decreasing survival and increasing apoptosis of prostate cancer cells (LNCaP) is significantly higher than that of free methotrexate not loaded in the nanoparticle [88].

Metastatic and invasive abilities are significant characteristics of malignant tumors. Thus, the scratch assay was performed to determine whether the knockdown of c-Myc expression by Se@BSA@Chi-DEC-MTX NPs could inhibit the migration behaviors of LNCaP prostate cancer cells. The combination of NPs and X-ray irradiation approaches significantly inhibits cell migration. On the other hand, treatment with NPs and X-ray irradiation exposure alone also has a certain inhibitory effect on cell migration. Suppression of cell migration in treated groups with Se@BSA@Chi-MTX NPs and Se@BSA@Chi-SCR-MTX NPs was almost equal, indicating that SCR had no effect on cell migration. However, the level of this inhibition was very high in the group treated with decoy ODNs and methotrexate nanoparticles, which can indicate the specific function of Myc decoy and its additive effect with methotrexate and X-irradiation exposure. Liao et al, confirmed that SeNPs may prevent the LNCaP cell migrations and invasions, which has been in line with our studies. They identified a series of microRNAs that may be upregulated considerably upon SeNPs treatment, among which miR-155-5p is a necessary compound in mediating the SeNP-inhibited migration and invasion of LNCaP cells, through directly targeting IκB kinase ε and Sma- and Mad-related protein 2 [89]. Furthermore, another study showed that the outcomes of the combination of nano-Se and radiotherapy on cell migration inhibition of cancer cells were better than in compare to use them alone [90].

## Conclusion

Molecular docking and molecular dynamics analysis showed the binding specificity and structure stabilities of the Myc/decoy complex, respectively. The characterization assays revealed that the designed nanostructure has suitable properties for drug delivery. The cell uptake test of MTX-targeted nanostructures on LNCaP cell line showed high absorption percentage. The MTX- targeted selenium nanostructures containing Myc-specific DEC along with X-irradiation exposure significantly decreased cell viability, induced apoptosis, arrested cell cycle phases, and eventually inhibited the migration and metastasis of cancer cells. Altogether, the Se@BSA@Chi-DEC-MTX nanostructure has the potential to be used as a combination therapy combined with radiation therapy in future *in vivo* and clinical studies by reducing the side effects of drugs.

## Supplementary Materials

**Table S1:** Size, PDI and Zeta potential of different nanoparticle formulations

Nanostructures	Size mean	PDI mean	Zeta mean
SeNPs	155.26	0.129	−0.849
	SD = 2.65	SD = 0.008	SD = 0.17
Se@BSA NPs	176.6	0.42	−9.56
	SD = 3.81	SD = 0.017	SD = 0.98
Se@BSA@Chi NPs	203.9	0.29	3.75
	SD = 0.32	SD = 0.016	SD = 0.64
Se@BSA@Chi-DEC NPs	214.73	0.26	−2.42667
	SD = 1.5	SD = 0.035	SD = 0.32
Se@BSA@Chi-SCR NPs	214.86	0.34	−2.26
	SD = 2.53	SD = 0.039	SD = 0.086
Se@BSA@Chi-DEC-MTX NPs	229.33	0.423	−9.27333
	SD = 0.713	SD = 0.008	SD = 0.273
Se@BSA@Chi-SCR-MTX NPs	231.1	0.408	−8.81
	SD = 1.52	SD = 0.007	SD = 0.296

## Data Availability

Supporting data related to this work are available upon request.

## List of Abbreviations

PC: Prostate cancer
PSA: Prostate-specific antigen
AR: Androgen receptor
TFs: Transcription factors
TFDs: Transcription factor decoy
siRNA: Small interfering RNA
MTX: Methotrexate
PSMA: Prostate-specific membrane antigen
SeNPs: Selenium nanoparticles
BSA: Bovine serum albumin
Chi: Chitosan
EDTA: Ethylenediaminetetraacetic acid
EDC: 1-Ethyl-3-(3-dimethylaminopropyl)carbodiimide
NHS: N-hydroxysuccinimide
Cy3: Cyanine-3
Se: Selenium
DEC: Myc decoy
SCR: Myc scramble
ODNs: oligodeoxynucleotides
3D-DART: 3DNA-driven DNA analysis and rebuilding tool
HADDOCK: High ambiguity driven protein-protein docking
TIP3P: Transferable intermolecular potential with 3 points
RMSD: Root mean square deviation
RoG: Radius of gyration
RMSF: Root mean square fluctuation
FTIR: Fourier transform infrared
KBr: Potassium bromide
UV-Vis: Ultra violet–visible spectrophotometry
DLS: Dynamic light scattering
PDI: Polydispersity index
EDX: Energy dispersive X-ray spectroscopy
TEM: Transmission electron microscope
SDS: Sodium dodecyl sulphate
PI: *Propidium iodide*
MD: Molecular dynamics
ROS: Reactive oxygen species
